# Non-genotoxic carcinogen exposure induces defined changes in the 5-hydroxymethylome

**DOI:** 10.1186/gb-2012-13-10-r93

**Published:** 2012-10-03

**Authors:** John P Thomson, Harri Lempiäinen, Jamie A Hackett, Colm E Nestor, Arne Müller, Federico Bolognani, Edward J Oakeley, Dirk Schübeler, Rémi Terranova, Diana Reinhardt, Jonathan G Moggs, Richard R Meehan

**Affiliations:** 1 MRC Human Genetics Unit, Institute of Genetics and Molecular Medicine, University of Edinburgh, Western General Hospital, Edinburgh EH4 2XU, UK; 2 Investigative Toxicology, Preclinical Safety, Translational Sciences, Novartis Institute for Biomedical Research, Lichtstrasse 35, CH-4057 Basel, Switzerland; 3 Breakthrough Breast Cancer Research Unit, University of Edinburgh, Western General Hospital, Edinburgh EH4 2XU, UK; 4 Friedrich Miescher Institute for Biomedical Research, Maulbeerstrasse 66, 4058 Basel, Switzerland; 5 Biomarker Development, Human Genetics and Genomics, Translational Sciences, Novartis Institutes for Biomedical Research, Maulbeerstrasse 66, 4056 Basel, Switzerland; 6 Member of MARCAR consortium

## Abstract

**Background:**

Induction and promotion of liver cancer by exposure to non-genotoxic carcinogens coincides with epigenetic perturbations, including specific changes in DNA methylation. Here we investigate the genome-wide dynamics of 5-hydroxymethylcytosine (5hmC) as a likely intermediate of 5-methylcytosine (5mC) demethylation in a DNA methylation reprogramming pathway. We use a rodent model of non-genotoxic carcinogen exposure using the drug phenobarbital.

**Results:**

Exposure to phenobarbital results in dynamic and reciprocal changes to the 5mC/5hmC patterns over the promoter regions of a cohort of genes that are transcriptionally upregulated. This reprogramming of 5mC/5hmC coincides with characteristic changes in the histone marks H3K4me2, H3K27me3 and H3K36me3. Quantitative analysis of phenobarbital-induced genes that are involved in xenobiotic metabolism reveals that both DNA modifications are lost at the transcription start site, while there is a reciprocal relationship between increasing levels of 5hmC and loss of 5mC at regions immediately adjacent to core promoters.

**Conclusions:**

Collectively, these experiments support the hypothesis that 5hmC is a potential intermediate in a demethylation pathway and reveal precise perturbations of the mouse liver DNA methylome and hydroxymethylome upon exposure to a rodent hepatocarcinogen.

## Background

Methylation of the fifth carbon of a cytosine base (5-methylcytosine (5mC)) in the dinucleotide sequence CpG is a well-established epigenetic modification of vertebrate DNA thought to have important roles in the preservation of genomic integrity, allele-specific expression of imprinted genes, maintenance of X-chromosome inactivation in females, tissue-specific gene regulation and long-term silencing of genes and retrotransposable elements [1,2]. Until recently, incorporation of a methyl group was thought to be the only form of direct DNA modification in the mammalian genome. However, landmark studies by two groups in 2009 re-discovered the modified base 5-hydroxymethylcytosine (5hmC) in mouse purkinje cells and granule neurons [3,4], a mark initially found over 50 years ago in T2 phage [5]. Shortly after this work, it was shown that a group of enzymes belonging to the TET family (TET1,2 & 3) of Fe(II) and α-KG-dependent dioxygenases utilize molecular oxygen to transfer a hydroxyl group to 5mC to form 5hmC [4,6-9]. In human cancers, the TET genes were found to exhibit a substantial reduction in their expression levels with global loss of 5hmC in tumors relative to surrounding tissue [10]. Recently, several studies have focused on one of these enzymes, TET2. Not only was this enzyme found to be frequently mutated or inhibited in many human acute myeloid leukemias, but its inactivation correlates with a hypermethylation phenotype [11-13]. These observations fit with a mechanism whereby the deposition of 5hmC at promoters can subsequently lead to demethylation of DNA, in a dynamic cycle of DNA demethylation and re-methylation, perhaps mediated by DNA glycosylases [14,15]. In support of this, inhibition of TET1 function in embryonic stem cells also leads to accumulation of DNA methylation at CpG-rich regions [16-18].

The genome-wide patterns of 5hmC have been described for both cultured cells [16-25] and tissues [26-29] with the general consensus that 5hmC-marked DNA is enriched over the bodies of expressed genes as well as at enhancer elements. When dynamically present at CpG-abundant promoter regions 5hmC may function as part of a demethylation pathway that promotes a methylation-free state, possibly through base excision repair pathways [30,31]. Recent work investigating epigenetic reprogramming events in the mouse zygote support this hypothesis through the finding that the rapid active demethylation seen in the paternal pro-nuclei is accompanied by an accumulation of genome-wide 5hmC and its derivatives in the absence of cell division [6,32,33].

A reprogramming mechanism for DNA methylation may also underpin the molecular changes that occur during the development of non-genotoxic carcinogen (NGC)-induced carcinogenesis [34-36] via the mis-expression of genes that promote liver tumor formation [34,35,37-39]. Several NGCs directly regulate nuclear receptors, including the constitutive androstane receptor (*CAR*; also known as nuclear receptor subfamily 1 group I member 3 (*Nr1i3*)) and peroxisome proliferator activated receptor alpha (*Ppara*), which mediate the transcriptional regulation of enzymes involved in response to drug exposure [40]. Many nuclear receptors, including PPARγ, interact with the DNA repair protein thymine DNA glycosylase (TDG), which can potentially mediate DNA demethylation at target genes by base excision repair mechanisms [15,33,41,42].

In a recent study, we reported that liver-specific changes at the DNA methylation level occur in a subset of mouse gene promoters following 28-day exposure to the well-studied NGC phenobarbital (PB) [43]. Locus-specific changes in histone modifications and loss of 5mC was observed at some of these promoter regions, which was coupled to an increase in the transcriptional activity of associated genes. Together this suggests that PB exposure can transduce an epigenetic switch from a repressive to an active chromatin state at selected target genes. Here, we map on tiled arrays the 5mC and 5hmC genomic patterns in both control and 28-day PB exposed mouse livers to examine the dynamic relationship between these two marks and their perturbation upon NGC exposure. In addition, we performed genome wide chromatin immunoprecipitation (ChIP) sequencing (ChIP-seq) to investigate PB-induced changes of three histone modifications, H3K4me2 (histone H3 lysine 4 di-methylation), H3K27me3 (histone H3 lysine 27 tri-methylation) and H3K36me3 (histone H3 lysine 36 tri-methylation). Our hypothesis is that changes in 5hmC profiles may be associated with PB-induced transcriptional remodeling in the liver. We find that chromatin modifications and the profiles of 5hmC and 5mC are pharmacologically perturbed over a subset of genes in a transcription-associated manner following continuous 28-day PB exposure. Together, these integrated epigenomic and transcriptomic profiling data provide novel insight into the molecular responses to a rodent hepatocarcinogen and may ultimately underpin the identification of novel early biomarkers for NGCs.

## Results and discussion

### Genomic distribution of 5mC- and 5hmC-marked DNA in the mouse liver

A 5hmC DNA immunoprecipitation assay (HmeDIP) was carried out on DNA from groups of control and PB-treated animals (each n = 5) using a highly specific anti-5hmC antibody (Additional file 1a,b). Enrichment was validated using quantitative PCR (qPCR) at candidate loci previously identified as being marked by the 5hmC modification [26] (Additional files 2 and 3). The 5hmC-enriched fractions were then applied to a large-scale promoter tiling-array (Nimblegen 2.1M Deluxe Promoter Array) to generate a representative pattern of the 5hmC landscape in mouse liver. The same procedure was repeated on the same DNA samples with an anti-5mC antibody (methylated DNA immunoprecipitation (MeDIP)) [44], allowing a direct comparison of the two DNA modifications. The 28-day 5hmC and 5mC raw data files have been deposited with Gene Expression Omnibus (GEO series number [GSE40540]).

**Figure 1:**
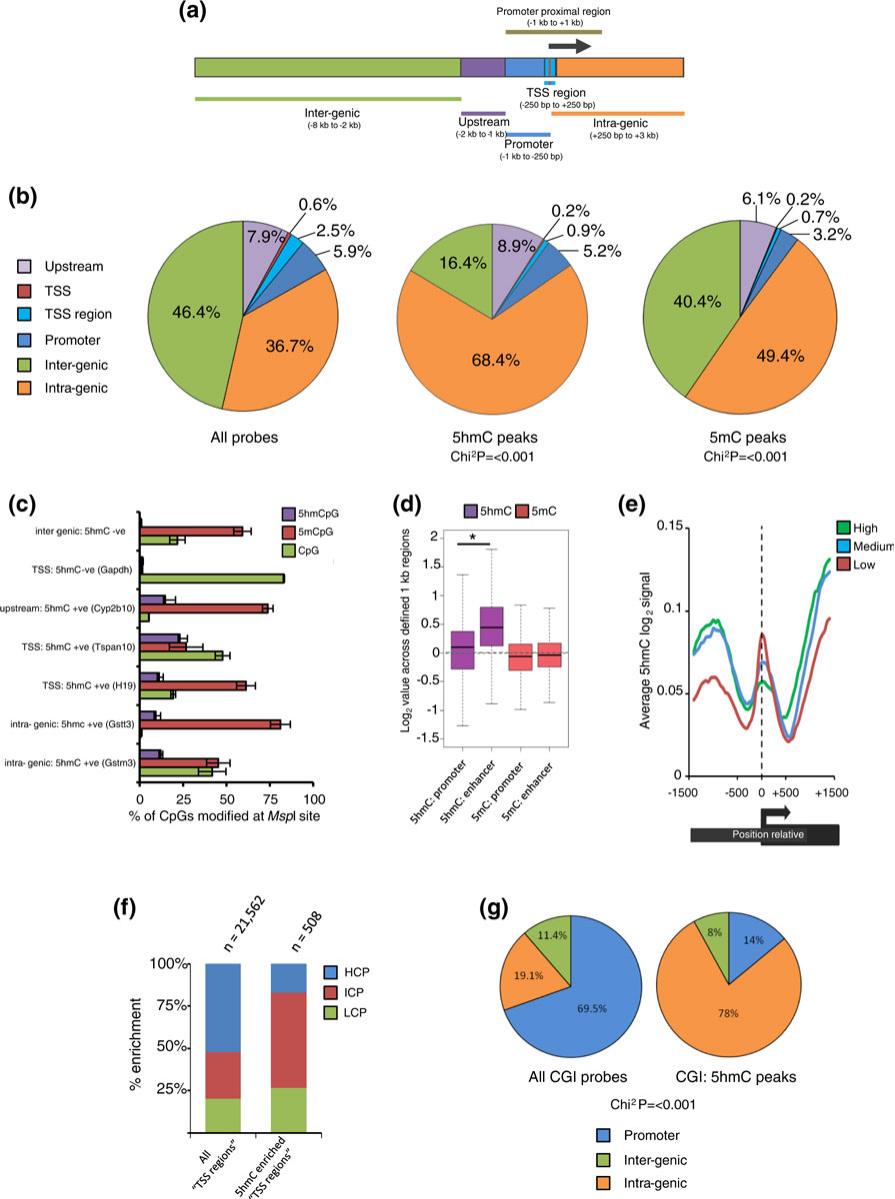
**5hmC profiling of mouse liver DNA**. **(a) **An 11 kb promoter array region split into six indicated regions for epigenetic mapping analysis. **(b) **5hmC and 5mC enrichment peaks in liver DNA map largely to intra-genic regions: left, distribution of all array probes; right, 5hmC and 5mC enrichment peaks. Chi^2 ^values indicate significance of the peak distributions compared to distribution of all array probes. **(c) **EpiMark qPCR of hmCpG (purple), 5mCpG (red) and non-modified CpG (green) levels over loci in control livers (n = 2). Percentage scores represent frequency of each CpG state over a single *Msp*I site. '5hmC +ve', 5hmC-positive regions; '5hmC -ve', 5hmC-negative regions. Error bars represent standard errors. **(d) **Box plot showing levels of 5hmC (purple) and 5mC (red) over 1 kb long enhancer and promoter regions. Asterisk denotes significant difference in signal levels (*P *< 0.001). **(e) **Sliding window analysis of average 5hmC profiles centered at genes' TSS regions based on their transcriptional activities. 5hmC levels differ over the TSS and flanking regions in a transcription-dependant manner. Highly transcribed genes (green) contain less 5hmC directly over the TSS and greater levels at flanking regions than medium (blue) and lowly expressed (red) genes. **(f) **5hmC-enriched TSS regions are largely associated with intermediate CpG content sequences (ICP; red). The CpG density of all TSS regions (left plot) reveals a skew towards high CpG content sequences (HCP; blue) over most promoters. In contrast, 5hmC-marked TSS regions tend to contain ICP promoters. LCP denotes regions of low CpG content. **(g) **Left: distribution of all probes associated with a CGI (n = 87,234). Right: distribution of a small number of CGI probes that overlap with 5hmC probes (n = 601). Chi_2 _values represent the significance of the 5hmC CGI peak distributions compared to distribution of all CGI probes.

To accurately determine genome-wide regions of 5hmC and 5mC enrichment, peak regions were identified (see Materials and methods) and assigned uniquely to one of six non-overlapping genic categories, according to their position relative to a nearby transcription start site (TSS) (Figure 1a). In total, 96,003 probes reside in 5hmC peaks and 47,876 probes in 5mC peaks across the 2,056,330 probes on the array (Figure 1b, left). Both the distribution of 5hmC and 5mC peaks differed significantly from the distribution of all the probes on the array (Chi^2 ^test *P *< 0.001; Additional file 4). In agreement with published data sets, the majority of 5hmC peaks were found to reside within gene bodies (68.4%; 56% intronic and 12.4% exonic) whilst only 6.3% of all peaks were found within promoter regions (-1 kb to +250 bp) (Figure 1b, middle). Similarly, there was enrichment for the 5mC peaks within gene bodies (Figure 1b, right). To quantify the absolute levels of both 5hmC as well as 5mC over these regions, we used the EpiMark™ 5hmC and 5-mC Analysis Kit (New England BioLabs) followed by qPCR (Figure 1c; Additional file 5; see Materials and methods). Overall, the average level of 5hmC across all loci tested was approximately 10% with no enrichment greater than 25% observed (Figure 1c) whilst average levels of both 5mCpG (approximately 48%) and non-modified CpG (approximately 37%) were considerably higher. In agreement with the peak-based analysis (Additional file 4), 5hmC levels were (rofl)low (<2% of CpGs) over both an inter-genic region on chromosome 7 as well the TSS region of *Gapdh *but enriched (10 to 15%) over two intra-genic regions (*Gstt3 *and *Gstm3*) and a region upstream of the *Cyp2b10 *promoter.

As studies have shown that 5hmC-modified DNA is particularly enriched at enhancer elements in cultured cells [19,22,23], we expanded our analysis to investigate such sites present on our array. Of the 23,556 probes covering defined enhancers on our array, 15.4% overlapped with peaks of 5hmC whilst only 1.5% overlapped with peaks of 5mC (Additional file 6). Extension of this analysis revealed that, on average, 1 kb long enhancer regions present on the array contained significantly more 5hmC than was found over the defined promoter regions (Figure 1d; *P*-value < 0.001) whilst no such difference was observed for the 5mC mark. Finally, as the array does not contain repetitive DNA, we also tested these regions directly by standard qPCR to determine the relative enrichment of both 5hmC and 5mC over major satellites, LINEs and intracisternal A-particle (IAP) elements (Additional file 7). As expected from previously published work [18,28] major satellites, LINEs and IAP elements are enriched for 5mC, but not 5hmC, confirming that the 5hmC genomic fraction is limited to non-repetitive regions.

### 5hmC enrichment at promoters and gene bodies is linked to transcriptional state

Recent studies have revealed that the levels of 5hmC over promoters and within gene bodies correlate with transcriptional activity in embryonic stem cells [16,17,20,21] and both human and mouse tissue [26-29]. To test this for the first time in the mouse liver, average 5hmC profiles were plotted around the TSS and flanking regions (TSS ± 1.5 kb) of genes with high, medium and low levels of expression (Figure 1e). On average, promoters associated with low levels of gene expression marked with higher levels of the 5hmC modification directly over the TSS than was found over the promoters of highly expressed genes, indicating that the distribution of promoter 5hmC in the mouse liver is associated with the relative levels of transcriptional activity. Although the majority of the probes on the array map specially to promoter regions, a series of short genes (n = 775, <3 kb in total length) were also covered in their entirety, which allowed for the analysis of 5hmC patterns through the bodies of genes. The distribution of 5hmC at the TSS of this subset of short genes is consistent with the genome-wide distribution, whilst an enrichment of 5hmC is seen in the body of these genes in a transcription-associated manner (Figure 1e; Additional file 8).

### 5hmC is enriched at the TSS of a subset of intermediate CpG promoters

Although the majority of genes reveal depletion of 5hmC in the regions surrounding their TSS, a subset (n = 508 genes) was found to contain an enrichment of the modification over these regions (Additional files 9 and 10). Independent verification by 'EpiMark' qPCR revealed that the levels of 5hmC enrichment at two unique TSS regions (*H19 *and *Tspan10*) surpass those observed over the upstream and intra-genic regions tested (Figure 1c). Based on expression profiling data, these genes exhibit lower levels of transcription than the average of all genes on the array (Additional file 11), which is in agreement with the earlier observation that genes with higher levels of 5hmC over their TSS tended to be lowly expressed (Figure 1e; Additional file 8). Furthermore, genes with 5hmC-marked TSS regions also contain a slight but significant (Fisher's exact test, *P *< 0.001) enrichment for genes involved in tissue-specific patterns of expression (Additional file 12). In agreement with earlier studies, sequences associated with 5hmC-marked TSS regions were largely (56.5%) found to be of an intermediate CpG density (these sequences are termed ICPs; 1 to 5 CpGs per 100 bp; Figure 1f) [16,21]. Interestingly, the 5hmC-enriched TSS regions are also marked with higher levels of 5mC than is found at all genes (Willcox test, *P *< 0.001; Additional file 11). Although the biological relationship between CpG density and 5hmC levels is unclear, it may reflect the fact that CpG-rich regions tend to be largely maintained in a non-modified state (such as at CpG islands; 'CGIs') whilst ICPs are often methylated in a tissue-specific manner [45].

### CpG islands marked by 5hmC tend to be non-promoter-associated

As CpG density appears to be important in the marking of promoter regions with the 5hmC modification, the CGIs covered on the array (16,002) were analyzed for their association with peaks of 5hmC. Although the majority of CGIs are largely non-methylated, a subset did contain at least one peak of 5hmC (601 peaks aligned to CGIs). Of these, the vast majority (78%) were found to correspond to intra-genic CGIs, which were not associated with promoter regions (Figure 1g; Additional file 13). It is possible that the intra-genic CGIs contain higher levels of the 5hmC mark simply due to the fact that they reside within the bodies of genes, which themselves are regions of 5hmC enrichment. As the regions upstream of CGIs (termed 'CGI shores') have been implicated as regions of differential methylation between tissues and cancers [46], we investigated regions 1 kb upstream of annotated CGIs. Similarly to the CGIs themselves, we find no strong enrichment in both the 5hmC and 5mC marks at these loci (Additional file 14).

### 5hmC-marked regions are associated with an active chromatin state

**Figure 2:**
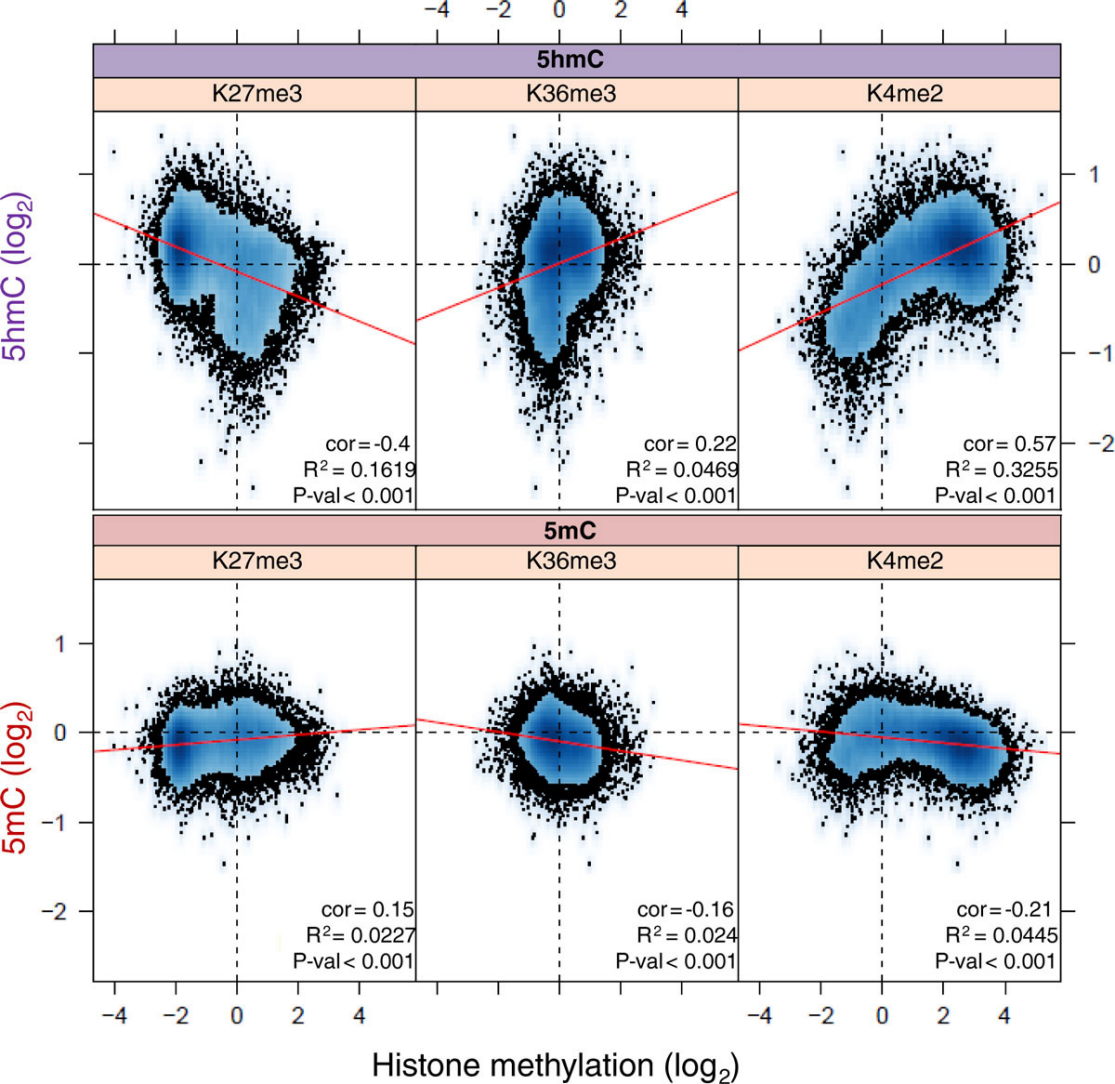
**Analysis of relationships between DNA and histone modifications in control mice livers**. Density scatter plot calculated by plotting the average promoter 5hmC or 5mC log2 score (y-axis) against either the average associated gene body H3K27me3 log2 value, gene body H3K36me3 log2 value or promoter H3K4me2 log2 value (x-axis). Trend lines (red) highlight correlations between the data sets (with associated R_2 _and Pearson correlation: 'cor' values). The density of genes/promoters is indicated by the grade of blue, and data points at the periphery of the main data density are indicated by black dots.

The patterns of promoter and gene body H3K4me2, H3K27me3 and H3K36me3 levels were determined by genome-wide ChIP-seq profiling on control livers (n = 2) to investigate potential links to associated promoter 5hmC and 5mC levels (Figure 2). Average promoter H3K4me2 signals reveal a striking correlation with promoter 5hmC values (Pearson correlation = 0.57, *P*-value < 0.001); this correlation occurs to a lesser degree with gene body H3K36me3 signals (Pearson correlation = 0.22, *P*-value = 0.001). As these histone modifications are typically associated with euchromatic regions of the genome, this indicates that the 5hmC modification may be associated with active chromatin states over both the promoters and the bodies of genes. In addition, there was a strong anti-correlation between promoter 5hmC levels and gene body H3K27me3 signals (Pearson correlation = -0.4, *P*-value = < 0.001). Although promoter 5mC signals have far weaker correlations to the histone modifications, they are opposite to those observed for the 5hmC mark, indicating that these two marks are functionally distinct.

### Phenobarbital induces perturbations of 5hmC and 5mC at selective promoter regions in liver

The epigenetic landscape is highly dynamic and often found to be perturbed by xenobiotics, including NGCs [34,35,43,47]. We previously investigated the effects of 28-day exposure to the widely studied NGC PB on 5mC levels over promoter regions (TSS -800/+100 bp), and found that promoter 5mC levels were reduced over a small subset of PB-induced genes in the mouse liver [43]. Given the proposed role for the 5hmC modification as an intermediate in a demethylation pathway, we used the high coverage promoter arrays to investigate if 28-day exposure to PB alters 5hmC patterns globally and specifically over promoter proximal regions (PPRs; Figure 1a). To characterize chromatin dynamics and their perturbations upon PB exposure, we also performed ChIP-seq for H3K4me2, H3K27me3 and H3K36me3 histone modifications along with Affymetrix gene expression analysis on the same tissue samples.

**Figure 3:**
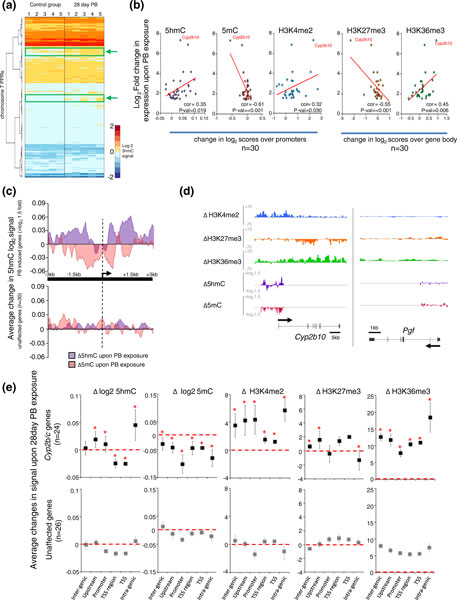
**Exposure to phenobarbital results in precise changes in the mouse liver 5hmC genomic profile**. **(a) **Heat map of average reproducible 5hmC levels over all PPRs on chromosome 7. PPRs are clustered by both 'Euclidian' and 'Ward' methods. Green boxes and arrows denote PPRs showing an increase in 5hmC levels in multiple PB animals. **(b) **Induction of gene expression is linked to a 5hmC increase and 5mC decrease over promoter regions. Scatter graph plots indicate average changes in 5hmC, 5mC, H3K4me2, H3K27me3 and H3K36me3 against fold change (>1.5) in expression for all genes upon PB treatment. Trend lines are displayed in red with associated Pearson correlation ('cor') values. **(c) **Top: plot showing reciprocal PB driven changes to 5hmC and 5mC patterns; most of which occur outside of the core promoters of PB-induced genes. Average changes in 5hmC levels upon PB treatment are shown in purple and changes in 5mC in red. Overlap in the changes of both marks results in darker regions. Bottom: plot of 30 genes showing no change in expression. **(d) **Patterns of DNA and histone modification change over the *Cyp2b10 *gene upon PB exposure. Genes unaffected by PB (for example, *Pgf*) do not display dynamic changes. Patterns of changes in 5mC (red), 5hmC (purple), H3K36me3 (green), H3K27me3 (orange) and H3K4me2 (blue) are plotted. ChIP-Seq samples were plotted on a scale of +70 to -70 reads; promoter arrays (5hmC and 5mC) plotted from +1.5 log_2 _to -1.5 log_2_. Gene structure with TSSs denoted by black arrows is shown below with scale bars. Dynamic and reciprocal changes in 5hmC and 5mC levels occur over regions flanking the TSS (+230 bp to +1,920 bp and -1,470 bp to -1,760 bp) and a long-range upstream element (-7,720 to -5,915 bp). **(e) **Average changes in epigenetic marks over the *Cyp2b/2c *gene family compared to genes unaffected by 28-day PB exposure. Average changes in the log_2 _scores (DNA modifications) or fold change in number of reads (histone modifications are plotted against regions outlined in Figure 1). Error bars are standard error and points showing significant deviation from unaffected genes (Willcox test, *P*-value < 0.005) are denoted by the asterisk. The red dashed line represents zero change in epigenetic marks upon PB exposure.

Globally we find that the majority of genes do not undergo any significant change in expression upon PB exposure and this was reflected in the fact that both DNA and histone modifications were also largely un-altered across the majority of PPRs (Figure 3a; Additional files 15, 16, 17, and 18). Furthermore, analysis of the enhancer elements present on the array also revealed no change in both 5hmC and 5mC modified DNA at these loci following drug treatment (Additional file 19). Although the majority of promoter proximal regions do not reveal dramatic changes in their epigenetic state upon PB exposure, select PPRs do show reproducible perturbations in 5hmC levels across multiple individual livers, albeit at relatively low levels (Figure 3a, green boxes/arrows).

To better understand the dynamics of 5hmC and 5mC levels across PPRs following PB-induced gene activation, the changes in the DNA and histone modifications were plotted against 30 genes that did show a clear increase in their expression levels following 28-day PB exposure (>log_2 _1.5-fold induction; Figure 3b; Additional file 16, red boxes). This analysis revealed a relationship between a gain of 5hmC levels (Pearson correlation = 0.35, *P*-value = 0.019) over the PPRs of induced genes along with a loss of 5mC (Pearson correlation = 0.61, *P*-value = < 0.001). This reciprocal gain in 5hmC/loss in 5mC also corresponds to a general change in the chromatin configuration over these induced genes with increases seen in PPR H3K4me2 levels (Pearson correlation = 0.32, *P*-value = 0.030) and gene body H3K36me3 levels (Pearson correlation = 0.45, *P*-value = 0.0063). In contrast, gene body levels of the H3K27me3 modification, often associated with silencing events [43,48,49], are reduced at many PB-induced genes (Pearson correlation = -0.55, *P*-value < 0.001). As a control, the relationships between these marks and the expression levels of 30 genes that exhibited no transcriptional change following PB exposure revealed far lower Pearson correlation scores and no significant *P*-value scores (Additional file 20). From this analysis we conclude that an epigenetic switch takes place at the PPRs of genes activated by 28-day PB treatment in liver. Typically, PB induction of gene expression is accompanied by a loss of promoter 5mC, with an associated gain in promoter 5hmC levels (Additional file 21), which may represent an intermediate in a demethylation pathway.

To more accurately determine where the changes in both 5hmC and 5mC occur over the PPRs of the PB-induced genes, the average signal changes were plotted relative to the TSS over a ±3 kb window. The induced genes reveal a striking pattern of 5mC loss over the entire region, as well as a strong enrichment in 5hmC signal (Figure 3c, top panel). The changes in the two modifications were often seen to directly oppose each other, which may represent a replacement of the 5mC modification by the 5hmC form. Although regions outside of the core promoter experience a large increase in 5hmC, the regions surrounding the TSS show both a dramatic loss in both 5hmC and 5mC levels. As the promoters of active genes are typically non-methylated, this may represent a complete demethylation event. In contrast, the promoter regions of unaffected genes do not reveal any significant change in either mark upon PB exposure (Figure 3c, lower panel). It will be important through subsequent work to evaluate the functional significance of these changes in both 5hmC and 5mC over the regions that span the core of the promoter.

### PB treatment leads to dynamic transcriptional and DNA methylation (5mC/5hmC) changes at xenobiotic metabolism genes in liver

To better understand the functions of PB-induced genes, Gene Ontology term analysis was carried out on all genes with >1.5-fold increase in expression upon PB exposure (n = 30). This revealed enrichment for genes involved in xenobiotic metabolism (Additional file 22), including those encoding cytochrome P450s and glutathione S-transferases, as described previously for PB exposure [43,50]. Both of these gene families are involved in the detoxification of electrophilic compounds, including carcinogens [51-53]. CAR plays an essential role in phenobarbital-induced hepatocarcinogenesis in rodents [54]. Although *Cyp2b *gene induction is a ubiquitous downstream effect of CAR activation in rodent liver, and occurs in parallel to increased cell proliferation, it is uncertain whether increased cytochrome P450 enzyme activity itself plays a direct role in hepatocarcinogenesis [55]. Nevertheless, *Cyp2b10 *mis-expression is observed in a subset of liver tumors that occur after the initial inducer compound has been withdrawn or arose in the absence of exposure to PB [54,56,57]. Liver tumors that are glutamine synthetase-positive and mutated in β-catenin show concomitantly elevated *Cyp2b10 *expression [58]. We previously reported that the promoter region of *Cyp2b10 *is hypomethylated and associated with strong transcriptional induction following 28-day treatment with PB [43]. Here we find that the promoter region not only becomes hypomethylated (Figure 3d, red) upon PB treatment, but that these regions display a reciprocal increase in the levels of 5hmC (Figure 3d, purple). These reciprocal changes are also seen outside of promoter regions as far as 7 kb upstream of the TSS and 2 kb downstream (Figure 3d). In addition, the chromatin environment around this locus is dramatically altered upon PB exposure with histone marks conducive to gene activation events, such as promoter H3K4me2 (Figure 3d, blue) and gene body H3K36me3 (Figure 3d, green), increasing over the *Cyp2b10 *locus, whilst gene body H3K27me3 levels decrease (Figure 3d, orange). This locus appears to be one of the most dynamic regions in terms of 5hmC, 5mC and histone modification changes, and taken together with the finding that this gene shows the largest increase in gene expression, may correspond to the catalytic conversion of 5mC to 5hmC as part of a potential demethylation process.

To further investigate where the changes in the levels of 5hmC, 5mC and histone modification occur over the *Gst *and *Cyp2b *and *2c *families, average changes for these marks were calculated across the genomic regions outlined earlier (Figure 1a) and compared to genes displaying no change in gene expression upon PB exposure (Figure 3e). Using this approach we found significant increases in 5hmC levels at the upstream, promoter and gene body regions of the two gene families, with the most striking examples of epigenetic change observed over the *Cyp *family of genes (Figure 3e; Additional files 23 and 24). Through this analysis we discovered that the largest perturbation of the 5hmC mark occurred at the intra-genic regions of the *Cyp2b *and *2c *genes (36-fold enriched compared to gene body 5hmC levels over a similar number of genes unaffected by PB exposure, Willcox test, *P*-value 2.44E-10). Additionally, there was a significant reduction in both 5hmC and 5mC levels over the DNA around the TSS, which may represent a total demethylation event (Willcox test, *P*-value = 5.37E-06; Figure 3b). Analysis of the histone modifications changes over these two gene families revealed that H3K4me2 levels were increased across the upstream, promoter and intra-genic regions of both families upon PB treatment, whilst H3K27me3 levels were reduced over the promoters of both families and strongly reduced over the bodies of the *Cyp2b *and *2c *genes. Finally, PB-induced increases in H3K36me3 levels were largely observed over the upstream and intra-genic regions of the *Cyp2b/2c *and *Gst *genes. Together, these data reveal extensive pharmacologic perturbation of the mouse liver epigenome by a non-genotoxic carcinogen and identify reciprocal changes in 5mC and 5hmC patterns over the promoter regions of a subset of genes induced by PB.

### Prolonged stimulation by PB reciprocally perturbs 5hmC and 5mC patterns at the *Cyp2b10 *promoter, resulting in a demethylation event

**Figure 4:**
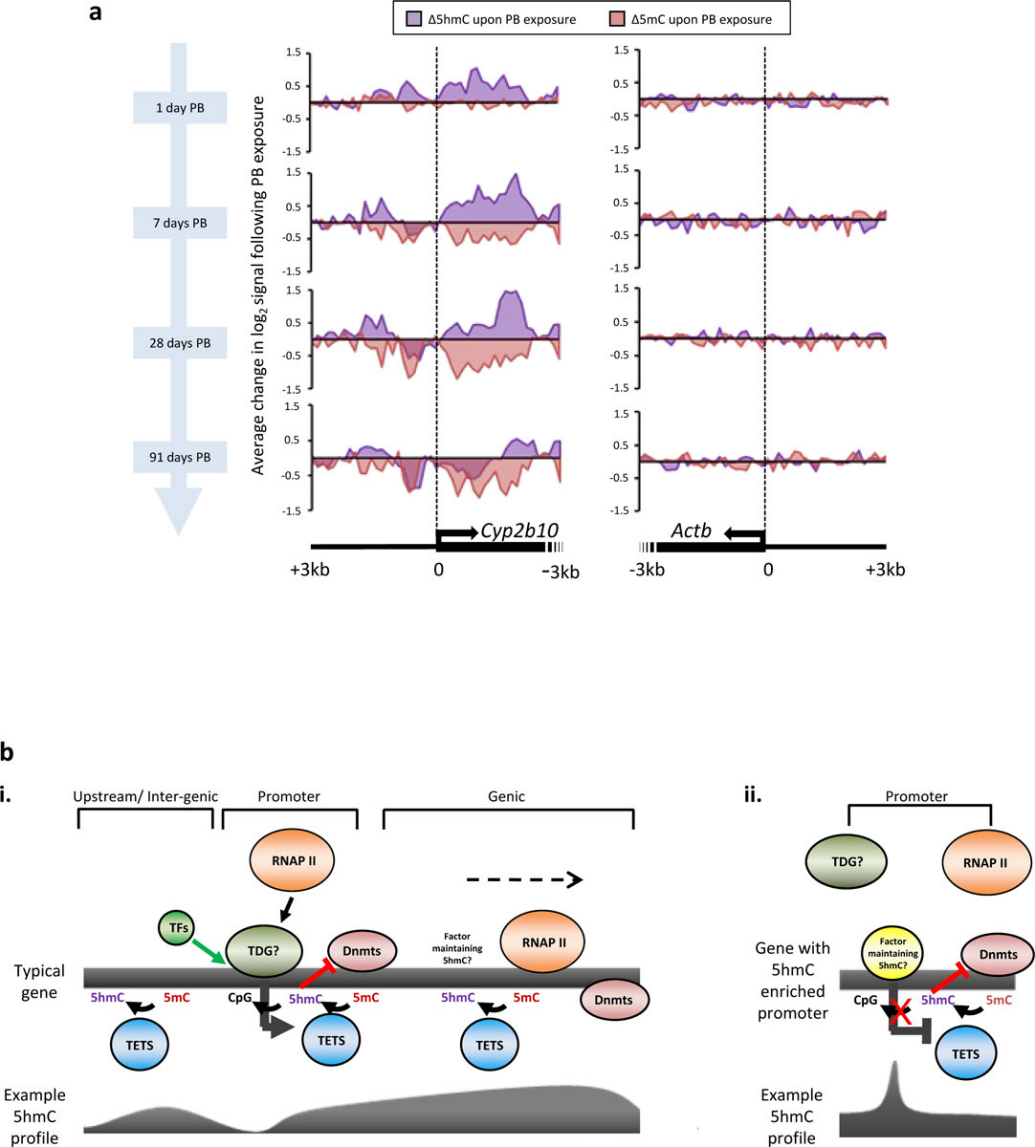
**Prolonged phenobarbital exposure results in depletion of both 5mC and 5hmC over the *Cyp2b10 *promoter**. **(a) **Continuous exposure to PB leads to reciprocal perturbations of 5hmC and 5mC patterns over the *Cyp2b10 *promoter (TSS ±3 kb). Mice receiving PB for 1, 7, 28 or 91 days display dynamic changes to their 5mC (red) and 5hmC (purple) profiles at *Cyp2b10*. Plots display changes in log2 score for either 5hmC or 5mC between PB-treated and control mice. 5hmC levels increased following 1 day of drug exposure whilst 5mC levels decreased with prolonged exposure. The region around the TSS lost both marks at around 7 days, which is most pronounced in mice that have received PB for 91 days. This may represent a transition to unmodified cytosine through a 5hmC intermediate. The *ActB *promoter exhibits no such dynamic change. **(b) **Models describing maintenance of 5hmC levels at expressed and non-expressed genes with example profiles for 5hmC displayed below (purple). Typical gene promoters (CGI) lack 5hmC- and 5mC-modified DNA (i). Demethylation is likely maintained by the Tet1 protein (5mC > 5hmC) and an unknown factor, possibly TDG, as part of the base excision repair pathway (5hmC > > C). 5hmC may prevent re-methylation occurring by inhibiting DNA methyltransferases (for example, Dnmt1). As 5hmC levels are high in the bodies of actively transcribing genes, Tet proteins must access this DNA, possibly in tandem with the elongating polymerase complex. A few promoter regions are enriched for 5hmC (ii), and associated genes tend to be inactive or lowly expressed (lacking the binding of RNAPII). Whether a unique factor is required to attract the Tets or repel TDG at these loci is unknown. TF, transcription factor.

As the promoter of the *Cyp2b10 *gene displayed particularly dramatic changes in both 5mC and 5hmC signals following 28-day PB exposure, we wished to investigate this perturbation following short-term PB dosing (1 day, 7 days PB treatment) and longer duration drug treatment (91 day exposure). At all time points tested both the 5hmC and 5mC patterns reveal striking reciprocal changes following PB exposure (Figure 4a), in keeping with earlier observations (Figure 3c). It was noted that prolonged drug treatment (91 day exposure) resulted in the loss of both 5mC and 5hmC from the core of the promoter region. Therefore, prolonged stimulation of the drug response gene *Cyp2b10 *by PB appears to result in the generation of an unmethylated CpG island through a 5hmC intermediate, which would facilitate high levels of expression at this locus. Further work investigating the perturbations to the transcriptome and epigenome following shorter doses of PB would elucidate the mechanisms of epigenetic change prior to and following activation of gene expression.

## Conclusions

The re-discovery of DNA modified by 5-hydroxymethylation in mammalian cells has changed the way in which we view the mammalian epigenome [3,4]. Here we present the first study of 5hmC patterns in the mouse liver and compare these to both 5mC patterns and associated gene transcription. Additionally, we report, for the first time, perturbations in 5hmC patterns over a set of genes that are induced upon exposure to PB, a well characterized rodent non-genotoxic carcinogen. Our results suggest that liver DNA has a similar but distinct distribution of 5hmC to that of embryonic stem cells [16-18,20,21] and mouse cerebellum tissue [26,28]. We also observe a correlation between the levels of the 5hmC modification and gene expression levels. We hypothesize that this may be due either to the modification of gene body 5mC marks to facilitate transcription by allowing the progression of polymerase complex or to stabilization of open chromatin by repelling methyl-CpG binding proteins and the maintenance methyltransferase Dnmt1 [4,59]. As such, this implies that distinct tissue-specific patterns of 5hmC are partly dependent on transcriptional differences.

Upon 28-day exposure to PB a group of genes (approximately 300) exhibited small but significant changes in gene expression whilst a subset of these (n = 30), typically with roles in drug response, were highly induced. Here we show that there is a strong correlation between increasing levels of 5hmC and decreasing levels of 5mC over the promoters of highly induced genes. Furthermore, these induced genes show characteristic changes in histone marks representing a move to a euchromatic state, which would facilitate transcription. The observed changes in the levels of promoter 5hmC and 5mC reveal that they are frequently reciprocal and tend to occur outside of the core promoter (Figure 3c). Regional differences in promoter 5mC levels have also been noted by others during differentiation [60]. Previous work reveals that, upon 28-day PB exposure, the 5mC modification is significantly reduced over regions spanning the TSS of the *Cyp2b10 *gene [43]. In this study, we expand upon this observation to reveal that although dynamic changes in both 5hmC and 5mC are occurring over promoter proximal regions, both marks are lost over regions surrounding the TSS, representing complete demethylation. Furthermore, this loss is all the more striking following prolonged drug exposure (91-day PB treatment; Figure 4a). This result implies that TSS regions may be bound by specific factors capable of completing the demethylation pathway (from 5mC via 5hmC to C). These factors may largely be promoter specific as the majority of 5hmC is seen outside these regions, residing within the bodies of actively transcribed genes (Figure 4b). It is possible that the transcriptional machinery itself can in some way maintain the 5hmC levels in these genic regions; however, although studies have shown that transcriptional elongation efficiency is high in DNA marked by 5hmC [61], the direct relationship between the transcriptional machinery and the maintenance of the 5hmC mark remains untested. Aside from the replication machinery, a strong candidate for a promoter-specific factor capable of removing the 5hmC would be TDG, which is able to directly remove the newly formed 5-hydroxymethyluracil by base excision repair [30,62] (Figure 4b). This would ultimately result in the conversion to a non-modified cytosine base (Figure 4a). Alternatively, further oxidation of 5hmC to 5-formylcytosine or 5-carboxylcytosine may provide suitable substrates for demethylation to non-modified cytosine via TDG-coupled base excision repair [9,15,33]. Although MBD4 and TDG could potentially remove 5-hydroxymethyluracil resulting from deamination of 5hmC, it has been recently shown that AID/APOBEC deaminases have substantially reduced activity toward 5hmC-containing templates compared to 5mC-containing counter parts, which would appear to rule out this pathway [63,64]. TDG has also been shown to play a critical role in the regulation of transcription through its interaction with transcription factors, nuclear receptors and the histone acetyl-transferases Crebbp and Ep300 [42,65,66]. The potential targeting of TDG through its interaction with nuclear receptors such as CAR may account for the locus-specific changes in 5hmC that occur in concert with PB-induced expression changes in liver (Figures 3c and 4a). It will be of interest to study this further in CAR null mice and mutants that contain the human CAR [67].

In conclusion, changes in 5hmC- and 5mC-modified DNA upon transcriptional activation by PB may represent an intermediate step in a demethylation pathway resulting in the presence of unmodified CpGs over core promoter regions. Further analysis of the dynamic changes in epigenetic marks associated with early transcriptional responses to PB, their long-term plasticity and status in PB-induced liver tumors will lead to a greater understanding of mechanisms of non-genotoxic carcinogenesis. Ultimately, if our observations are replicated in other NGC exposure models, this may lead to the identification of candidate epigenetic biomarkers for enhanced cancer risk assessment.

## Materials and methods

### Ethics statement

This study was performed in conformity with the Swiss Animal Welfare Law and specifically under the Animal Licenses No. 2345 by 'Kantonales Veterinäramt Basel-Stadt (Cantonal Veterinary Office, Basel) and No. 5041 by 'Kantonales Veterinäramt Baselland' (Cantonal Veterinary Office, Basel Land).

### Animal treatment and sample preparation

Male B6C3F1/Crl (C57BL/6 ♂ × C3H/He ♀) mice 29 to 32 days old were obtained from Charles River Laboratories (Germany). Animals were allowed to acclimatize for 5 days prior to being randomly divided into two treatment groups of five animals each. 0.05% (w/v) PB (Sigma-Aldrich, St Louis, MO, USA) was administered to one group through *ad libitum *access to drinking water for either 1, 7, 28 or 91 days. Mice were checked daily for activity and behavior and sacrificed on the last day of dosing depending on the dosage group. Livers were removed prior to freezing in liquid nitrogen and -80°C storage.

### Dot blot analysis of 5hmC antibodies

DNA from mouse liver and PCR products containing C, 5mC and 5hmC [68] were spotted onto a positively charged nitrocellulose membrane and immobilized with 0.4 M NaOH using a dot blot apparatus (Harvard Apperatus, Edenbridge, UK). The membranes were probed with anti-5mC monoclonal antibody 1.6 μg/μl (Diagenode, Liege, Belgium) diluted 1:4,000 in tris-buffered saline (TBS) and Western Blocking reagent (Roche Diagnostic, Mannheim, Germany), or with the anti-5hmC polyclonal antibody diluted 1:5,000 (Active Motif, La Hulpe, Belgium) followed by anti-rat horse radish peroxidase (Cell Signalling Technology, Boston, USA) and anti-mouse horse radish peroxidase (Sigma-Aldrich, Dorset, UK) prior to exposing the membrane to Image Quant (Image Quant LAS 4000; GE Healthcare, Bucks, UK). A duplicate membrane was produced for the DNA loading control and probed with anti-single stranded DNA 0.2 μg/μl (Demeditec Diagnostics, Kiel-Wellsee, Germany) diluted 1:3,000 raised against rabbit.

### HmeDIP and MeDIP protocol

Genomic DNA from mouse liver tissue samples was extracted by overnight proteinase K digestion (Sigma) in lysis buffer (50 mM Tris-HCl pH 8.0, 10 mM EDTA pH 8.0, 0.5% SDS) prior to phenol-chloroform extraction, ethanol precipitation and RNaseA digestion. Genomic DNA was sonicated (Bioruptor, Diagenode) to produce DNA fragments ranging in size from 300 to 1,000 bp, with a mean fragment size of around 500 bp. Fragmented DNA (4 μg for HmeDIP and 6 μg for MeDIP) was then denatured for 10 minutes at 95°C and immunoprecipitated for 3 h at 4°C either with 1 μl of rabbit polyclonal antibody against 5-hydroxymethylcytosine (Active motif, La Hulpe, Belgium; cat#39769) or with 15 μl mouse monoclonal antibody against 5-methylcytidine (Eurogentec, Seraing, Belgium; #BI-MECY-1000) in a final volume of 500 μl immunoprecipitation (IP) buffer (10 mM sodium phosphate (pH 7.0), 140 mM NaCl, 0.05% Triton X-100). This mixture was incubated with 60 μl of magnetic M-280 protein G Dynabeads (Invitrogen, Grand island, NY, USA; #100-03D) for 2 h prior to washing all unbound fragments three times with 1 ml IP buffer. Washed beads were then resuspended in 250 μl of lysis buffer and incubated with proteinase K for 2 h at 50°C. Immunoprecipitated DNA fragments were then purified by passing through DNA purification columns (Qiagen, Venlo, Netherlands) and eluting into 20 μl TE. For qPCR analysis, 10 μl were taken and diluted to 100 μl in TE with each qPCR reaction using 2 to 3 μl of diluted DNA. For microarray analysis, 10 μl of immunoprecipitated DNA was subjected to whole genome amplification (WGA) using the WGA2:GenomePlex Complete Whole Genome Kit (Sigma) and 6 μg of amplified material sent to Roche Nimblegen (Iceland) for Cy3 and Cy5 labeling and hybridization on 2.1M Deluxe mouse promoter tiling arrays.

### H3K4me2/H3K27me3/H3K36me3 native ChIP for genome-wide sequencing

Frozen mouse liver (150 mg) was isolated and ground into a fine powder using with Covaris Cryoprep (Covaris Inc., woburn, Massachusetts, USA). Nuclei were isolated by centrifugation through a 1.2M sucrose cushion prior to micrococcal nuclease (Sigma) fractionation into primarily mono- and di-nucleosomal fragments. For each histone mark, 100 μg of chromatin was used and 10% of the input taken prior to immunoprecipitation.

The immunoprecipitation, washes and DNA purification were done with Magna ChIP™ A Chromatin Immunoprecipitation Kit (Millipore, Billerica, Massachusetts, USA; #17-610) following the manufacturer's protocol

DNA (0.9 μg) for the input samples and 0.018 μg for the K4 and K27 samples were end-repaired and ligated to Illumina genomic DNA adapters according to the manufacturer's protocol (Illumina, 2010). The samples were purified using Agencourt AMPure XP beads (Beckman Genomics, Danvers, Massachusetts) and then subjected to 12 cycles of PCR as per the manufacturer's protocol (Illumina, 2010). No sample indexing was performed. Their size distributions were checked on an Aglient Bioanalyzer (Agilent). Each sample was then loaded as a 6pM solution on an Illumina GAIIx v7 paired-end flowcell using a cluster station instrument (Illumina). The flowcell was then subjected to 2 × 55 bp of SBS chemistry v4 on an Illumina GAIIx instrument.

DNA (1 μg) for the L samples and 0.018 μg for the K36 samples was end-repaired and ligated to Illumina TruSeq adapters according to the manufacturer's protocol (Illumina, 2011). The samples were purified using Agencourt AMPure XP beads (Beckman Genomics) and then subjected to 12 cycles of PCR as per the manufacturer's protocol (Illumina, 2011). The samples were indexed using the Illumina TruSeq indexes and purified using Agencourt AMPure XP beads and their size distributions checked on an Aglient Bioanalyzer (Agilent). The indexed libraries were then quantitatively pooled and a 9pM solution of multiplexed library was denatured and loaded on an Illumina TruSeq SE v1.5 flowcell using a cBot instrument (Illumina). The flowcell was then subjected to 51 bp of SBS chemistry on an Illumina HiSeq2000 instrument using the Illumina Real Time Analysis 1.12 software. The base calling and sample demultiplexing was performed using the Illumina CASAVA 1.8 software.

### Affymetrix gene expression analysis

Affymetrix expression arrays were performed following the methods outlined in earlier work [43], except that the dataset was normalized by robust multichip average (RMA) techniques [69] and the *P*-value for the log2 fold change was calculated with the R/Bioconductor LIMMA package using a moderated t-statistic.

### Processing of Nimblegen promoter arrays

Nimblegen 2.1M delux mouse promoter arrays (mm9 build) contain 2,056,330 unique probes of 50 to 70 bp in length with approximately 50 bp spacing distributed over 21,562 tiled regions spanning 52,016 annotated TSS regions over 20,718 unique genes. In addition these arrays cover 15,969 annotated CpG islands over both promoter and 'non-promoter' (inter-/intra-genic). Signals for each probe of the 5hmC-enriched samples (Cy5 labeled) were compared to input samples (Cy3 labeled) to generate log2 (IP/Input) scores (fold changes). These log2 scores were then normalized to correct for both saturation effects within individual arrays by Loess normalization and between the arrays by scale normalization using the Limma package in R/Bioconductor [70]. In order to remove individual variability the mean probe values were calculated for both the control and PB exposed group. Subsequent analysis was carried out by comparing the differences between the mean control and mean 28-day PB data sets and were plotted as changes in the probe values in PB mice relative to control mice. Raw data for the reciprocal 5mC HmeDIP and MeDIP experiments were processed the same way as 5hmC but were normalized separately. The 28-day 5hmC and 5mC raw data files have been deposited with Gene Expression Omnibus (GEO series number [GSE40540]; 28-day 5hmC [GSE40537], 28-day 5mC [GSE40538], and 28-day expression [GSE40773]).

#### Nimblegen 2.1M mouse promoter array GPL14890

Two sets of data were created for each modification. The first contains the values for all 2,056,330 probes on the microarray and was used for all analysis techniques which did not require an association to a nearby gene. A second data set was created that links the probes to a nearby gene so as to include affymetrix expression data from the same mice. This gene list is reduced in the numbers of probes it contains (387,612) as gene names were associated to all probes ±1 kb of an annotated Refseq TSS.

### Bioinformatic analysis of datasets

#### 'Peak-based analysis' of 5hmC and 5mC

In order to better characterize regions of 5hmC and 5mC enrichment, peak finding was carried out across the data sets. Peaks were defined as regions containing at least four probes in a five probe window above the 90th percent score of the entire data set. Using these parameters, 96,003 peaks of 5hmC (representing 4.7% of all probes) and 47,876 peaks of 5mC (representing 2.3% of all probes) were identified. To ensure peak finding was returning acceptable results, peaks were compared to qPCR-validated control regions (chr7:149,763,602-149,763,942, covering the promoter of the *H19 *gene, which is positive for 5hmC and 5mC; and chr6:125,115,497-125,115,656, covering the promoter of *Gapdh*, which is negative for both marks). Peaks of both 5hmC and 5mC were then interrogated for their genomic locations and results plotted as pie charts along with the general distribution of all probes on the array.

#### Analysis of CpG densities over 5hmC-enriched TSS regions

To further investigate the hmC-enriched TSS regions, the DNA of such sites was extracted and the relative CpG densities calculated (as number of CpGs per 100 bp). The hmC-positive TSS regions were then ranked by these densities grouped by low CpG content (LCP; <1 CpG per 100 bp), ICP (1 to 5 CpGs per 100 bp) and HCP (>5 CpGs per 100 bp). Overlap between regions enriched in hmC and CGIs was carried out by crossing regions with peaks in hmC to CGIs. The total number of probes covering CGIs was 87,234, whilst the number of CGI probes enriched in hmC was 601. The distribution of CGI-positive hmC-positive probes was then plotted as a pie chart next to the distribution of all CGI probes.

#### Sliding window analysis

Sliding windows of hmC and 5mC profiles were characterized over both unique Refseq TSSs ±1,500 bp (n = 1,000) and 'small', 2 to 3 kb long, complete genes found on the array (n = 775). Sliding window analysis was carried out using tools on the University of Edinburgh's GALAXY sever [71]. Sliding window analysis plots the average signal taken from data files of interest (for example, mean hmC normal probe values) and slides across regions of interest (chromosome, start, stop) in user-defined steps (in base pairs). Expression data from control and PB-treated mouse livers were generated on Affymetrix expression arrays similar to [43], and divided into three groups depending on levels of transcription (low = bottom 25% expression levels, high = top 25% expression levels, medium = remaining genes). These groups were then crossed to regions of interest for sliding window analysis (for example, TSS ±1.5 kb regions ranked by expression). Sliding window analysis was carried out using a window size of 200 bp and with a step size of 50 bp and average signals plotted.

#### Analysis of the tissue specificity of genes with 5hmC-enriched TSS regions

Box plots were generated comparing classes of genes (including all the genes on the deluxe promoter array to all 5hmC-marked TSS region genes). The specificity of a gene's expression pattern was measured by using a method based on information theory outlined by Martinez *et al. *[72]. A low score (0) indicates that a gene is uniformly expressed, and a high score (6.2) indicates that it is expressed specifically in one tissue. A previously published brain-specific gene set and housekeeping gene set determined by serial analysis of gene expression (SAGE) are shown for comparison [73].

#### Preparation of ChIP-Seq data sets for analysis

Sequencing reads were mapped to the mouse genome (built mm9) using the bowtie software [74]. For marks H3K4me2 and H3K27me3 bowtie was run in paired-end mode; for H3K36me3 it was run in single-end mode. As we investigated the enrichment in predefined regions of the genome (promoter and gene body), no peak finding was performed. From the mapped paired-end data the genomic locations of the inserts were calculated (from the forward and reverse read pairs). For the single-end data genomic locations were directly extracted from each mapped read. Duplicated locations were removed, yielding a non-redundant library of mapped genomic fragments. The number of fragments overlapping with pre-defined genomic regions such as gene bodies or promoters (see above) was counted. Within each histone mark the fragment counts per region were normalized by the total number of fragments and scaled to the mean of the libraries. The counts for each mark in each region were further normalized by the counts for the matching background sample to generate log2 fold changes (scores) between IP and background to avoid bias in genomic context. We report the mean score per group (n = 2) per region and mark.

#### Interrogation of changes in epigenetic marks over PPRs upon PB treatment

The mean log2 probe values over a 2 kb window (TSS ±1 kb; PPRs) were calculated for the hmC, 5mC and H3K4me2 data sets over each gene for both control, PB exposed and changes observed in PB mice. The expression changes upon PB were represented as fold change expression relative to the control mice. Average H3K27me3 and H3K36me3 levels in control, PB-exposed mice as well as changes seen in PB mice were calculated over the entire gene body and related to associated promoter regions (see Additional file 21 for an example).

Initial analysis was carried out using the entire hmC or 5mC data set, plotting the average changes in promoter hmC levels against changes in gene expression. Subsequent plots for changes in promoter hmC, 5mC, H3K4me2 as well as gene body H3K27me3 and H3K36me3 only include genes with at least 1.5-fold induction in gene expression (n = 30). Trend lines were then calculated over the resulting scatter graphs along with R^2 ^values.

Regions spanning the promoters (±3 kb) of the PB-induced genes (>log_2 _1.5-fold) showing induction along with 30 PB 'unaffected' genes were then selected and sliding window analysis carried out to plot the average changes in 5hmC and 5mC over these loci (see above for more on sliding window analysis). Coordinates of the PB unaffected genes are available on request.

Plots of the average changes in hmC, 5mC, H3K4me2, H3K27me3 and H3K36me3 were then carried out across two families of genes as well as 26 genes unaffected by PB exposure. Average values for changes in the epigenetic modifications were calculated across the defined regions (Figure 1). Error bars represent standard errors and data points with significant *P*-values are represented by an asterisk. Coordinates of the PB unaffected genes are available on request.

#### Heat map analysis of PB-driven 5hmC perturbation

Average 5hmC levels were calculated as explained above for each PPR. These were then separated based on the chromosome of origin and then plotted either for each individual liver (five controls, five PB treated; Figure 3a) or for the average of these control or PB exposed livers (Additional file 15). Heat maps were drawn using R with colors taken using the Colour Brewer package ('RdYlBu') ranging from values of -2.5 to +2.5 with a 0.5 interval. PPRs were ordered by the first query (that is, WT in Additional file 15) and clustered by both Euclidian and Ward methods.

#### Analysis of enhancer elements

We took 38,112 regions defined as mouse liver enhancers from datasets generated by Yim Shen *et al. *[75]. Midpoints of enhancer regions were then taken and expanded to create 1 kb stretches of DNA sequence before restricting the dataset to those present on the 2.1M mouse deluxe promoter array used for 5hmC and 5mC analysis. This resulted in 23,556 probes that corresponded to 1.15% of all probes on the array. Overlap between 5hmC/5mC-enriched peaks was then carried out and any probe residing in a peak of a mark as well as an enhancer region was scored. Box plots of 5hmC/5mC levels were then calculated over the 1 kb array enhancers as well as 1 kb promoter regions (TSS to -1 kb upstream).

### 5hmC-sensitive restriction digest-qPCR

The EpiMark kit (New England BioLabs, Ipswich, Massachusetts, USA) was used to quantify absolute levels of 5hmC and 5mC between the control and PB mouse livers as well as DNA from mouse brain. For the full protocol see the manufacturer's instructions. Typically, 10 μg of genomic DNA was taken and half treated with T4- phage β-glucosyltransferase for 12 to 16 h at 37°C. Both the β-glucosyltransferase treated and untreated samples were then divided into three PCR tubes and digested with either Msp1, HpaII or left uncut for a further 12 to 16 h at 37°C. Samples were proteinase K treated for 10 minutes at 40°C prior to dilution to 100 μl final volume in H_2_0 and heating to 95°C for 5 minutes. qPCR was carried out on 5 μl (approximately 0.8 μg DNA) of each sample on a Roche Lightcycler 480 PCR machine. Relative enrichments of the modifications were then calculated following formulae provided by New England BioLabs.

### Primers

For primers used for qPCR validation and 'EpiMark' analysis please see Additional file 25.

### Data access

Raw and processed data have been deposited into the Gene Expression Omnibus (GEO series number [GSE40540]).

## Abbreviations

5hmC: 5-hydroxymethylcytosine; 5mC: 5-methylcytosine; bp: base pair; CAR: constitutive androstane receptor (Nr1i3); CGI: CpG island; ChIP: chromatin immunoprecipitation; GEO: Gene Expression Omnibus; H3K27me3: histone H3 lysine 27 tri-methylation; H3K36me3: histone H3 lysine 36 tri-methylation; H3K4me2: histone H3 lysine 4 di-methylation; HmeDIP: 5hmC DNA immunoprecipitation assay; IAP: intracisternal A-particle; ICP: intermediate CpG content; IP: immunoprecipitation; LINE: long interspersed nuclear element; MeDIP: methylated DNA immunoprecipitation; NGC: non-genotoxic carcinogen; PB: phenobarbital; PPR: promoter proximal region; qPCR: quantitative PCR; RMA: robust multichip average; TDG: thymine DNA glycosylase; TSS: transcription start site.

## Competing interests

The authors declare that no competing interests exist. Authors who work for Novartis Institutes for Biomedical Research (NIBR) confirm that this does not alter their adherence to all the Genome Biology policies on sharing data and materials.

## Authors' contributions

Conceived and designed the experiments: JM, HL, RT, JT & RM. Performed the experiments: JH, JT, DR, HL, AM, FB and RT. Analyzed the data: JT, AM and CN. Contributed reagents/materials/analysis tools: DS. Wrote the paper: JT and RM (edits by JM, RT, HL, DS, CN, AM and RM). All authors have read and approved the manuscript for publication.

## Supplementary Material

Additional file 1 **Figure S1**. **(a) **Dot blot comparing the specificities and sensitivities of two commercially available 5hmC antibodies. Antibodies were tested over a dilution gradient of genomic liver DNA (200 ng/μl to 2.5 ng/μl) as well as for their relative affinities against PCR products synthesized in the presence of either dCTP, dmCTP or d5hmCTP [68]. Although both antibodies can distinguish 5hmC from 5mC and non-modified C (see controls), only the polyclonal (pAB) α-rabbit antibody (Active motif) can detect 5hmC in genomic mouse liver DNA. It must be noted that the relative levels of 5hmC present in the control PCRs are far greater than that in the genomic DNA. **(b) **The HmeDIP technique is highly specific for 5hmC-modified DNA relative to unmodified or methylated DNA. qPCR was run on immunoprecipitated material that had been spiked with a 5hmC-rich DNA sequence. Enrichment of the 5hmC rich control DNA was only observed when immunoprecipitated with the 5hmC antibody and not with an antibodies against 5mC, non-modified C or IgG. Click here for file

Additional file 2 **Figure S2**. qPCR analysis on pre- and post-whole genome amplification (WGA) material introduced no bias upon amplification. Confirmatory qPCR showing hmC enrichment at Tex19.1 and *H19 *promoter regions (known hmC-enriched loci) alongside a 5hmC-negative control region (*Gapdh *promoter) both prior to and after WGA. 5mC enrichment was tested in a similar way using positive control regions of the *H19 *ICR and Cyp2b10 promoter regions and a negative ICR. Click here for file

Additional file 3 **Figure S3**. **(a,b) **Validation of microarray profiling by qPCR. Correlation between HmeDIP and MeDIP qPCR enrichment values (% IP/input) to log2 values seen on the promoter microarrays is presented for 5hmC (a) and 5mC (b). Trend line (red) and R_2 _values are also shown. Pearson correlation values ('cor') shown alongside represent the closeness of fit between qPCR enrichment values (Y-axis) and log2 scores taken from microarray data (X-axis). Loci used for validation of HmeDIP were as follows: upstream region, 5' and 3' promoter regions of *Cyp2b10*, promoter regions of *Tex19.1*, *Actb*, *Gapdh *and *H19*. Loci used for the validation of MeDIP are as follows: ICRs of *H19 *and *Gnas*, intron 1 of *Cyp2b10 *and promoter regions of *CSA*, *Pou3f4*, *Ccdc34*, *Tyms*, *Ccr1*, *Cbx2 *and *Tacr3 *(see primer list in Additional file 25). Click here for file

Additional file 4 **Figure S4**. Normalized peak finding data for 5hmC and 5mC based on data from Figure 1c. The number of peaks represented in Figure 1c for both 5hmC and 5mC were divided by the number of probes covering each region (Figure 1a,c) to remove coverage bias. **(a,b) **Overall there are no large changes in the distribution of 5hmC (a) and 5mC (b) enriched peaks upon normalization with the exception of an increase in upstream 5hmC and 5mC peaks and reduction of inter-genic 5hmC peaks. Click here for file

Additional file 5 **Figure S5**. Validation of 'EpiMark' quantitative analysis techniques reveals highly similar levels of the DNA modifications between individuals. **(a-c) **The values of 5hmCpG (a), 5mCpG (b) and CpG (c)were plotted between two biological replicates to test the reproducibility of the result. Loci tested were as follows: TSS regions of *Gapdh*, *H19 *and *Tspan10*, an intra-genic region of *Gstm3*, a region upstream of the *Cyp2b10 *promoter and an inter-genic region spanning chr7: 149709621-149709863 (see primer list in Additional file 25). All modified forms of CpG are reproducible between individuals over the six loci. Pearson correlation values ('cor') are shown alongside representing the reproducibility of results. Click here for file

Additional file 6 **Figure S6**. A subset of enhancer elements present on the promoter array overlap with peaks of 5hmC. Total number of probes that overlap with 1 kb long enhancer elements defined by Shen *et al. *[75] (grey bar) are plotted next to enhancer probes that also contain a peak of either 5hmC (purple bar) or 5mC (red bar). Overall, approximately 15% of enhancer probes on the array overlap with peaks of 5hmC. Click here for file

Additional file 7 **Figure S7**. qPCR analysis of 5hmC (purple) and 5mC (red) levels at repeat elements (major satellites, LINEs and IAPs) in liver DNA. Enrichment values are plotted as percentage IP relative to the input. Click here for file

Additional file 8 **Figure S8**. Sliding window analysis of 5hmC and 5mC over full-length genes (n = 775) covered on the array. Levels of 5hmC increase throughout the body of a gene in a transcription-dependant manner. Expression levels were calculated within the 775 'small' gene set. Top 25% = highly transcribed (green), bottom 25% = lowly transcribed (red). In contrast to 5hmC, levels of 5mC are higher over the TSS of lowly expressed genes and show less of a correlation with transcriptional levels throughout the gene body. Click here for file

Additional file 9 **Figure S9**. Examples of 5hmC-enriched TSS regions along with more typical 5hmC-depleted TSS regions. Two examples of 5hmC-enriched genes are shown on the left (*Rnas10 *and *Tmem139*). In comparison, a more typical (5hmC depleted) TSS region is shown on the right for *Bckdha *and *Exosc5*. All scales on the Y-axis are from log_2 _1.5 to -log_2 _1.5. Individual X-axis scale bars are represented by a black bar. The 5hmC profile is in purple and 5mC in red; the transcription start site (black arrow) and gene structure are also illustrated. Exons are shown as perpendicular lines. Click here for file

Additional file 10 **Table S1**. List of genes with a 5hmC-enriched 'TSS region' (n = 508). Click here for file

Additional file 11 **Table S2**. Average promoter 5hmC and 5mC log2 scores across all the genes on the array. Click here for file

Additional file 12 **Figure S10**. Genes with 5hmC enrichment over their TSS show some enrichment towards those that are expressed in a tissue-specific manner. The relative levels of tissue specificity were plotted (see Materials and methods; Si value of 0 = non tissue specific, Si value of 6.2 = highly tissue specific) against groups of genes with high tissue specificity as well as housekeeping genes. In comparison to all of the genes on the array there is a small but significant (*P*-value < 0.001) increase in the level of tissue specificity for genes with 5hmC-enriched regions spanning the TSS. The red lines represent the median score of the group of genes. The upper circles represent outliers of the majority of genes. Click here for file

Additional file 13 **Figure S11**. Examples of CGIs with high CpG density (HCP) that are enriched for 5hmC. Although the majority of probes covering CGIs do not contain peaks of 5hmC, those that do show a striking enrichment over certain loci. **(a-c) **The majority of the 5hmC-enriched probes mapping to CGIs are inter-genic (a,b) or intra-genic (c). Intragenic CGIs in (a) are found at chr9:101,153,320-101,154,414 (left panel) and chr11:90,279,237-90,279,648 (right panel). Intergenic CGIs shown in the example in (c) are found within the gene *Zip4*. **(d,e) **Promoters with CGIs (*Actb *is the example (d)) are also found to be largely depleted in the 5hmC modification in contrast to those that lack CGIs (*Gstm6 *is the example (e)). All scales on the Y-axis are from log_2 _+2 to -log_2 _2. Transcription start site (black arrow) and gene structure are illustrated below each example. Exons are shown as perpendicular lines. Scale bars are represented at the bottom right of each figure. Click here for file

Additional file 14 **Figure S12**. 5hmC and 5mC levels are not enriched over 'CGI shores'. The total number of probes found over regions 1 kb upstream of annotated CGIs were selected (upper panel and lower panel grey bar) and the number of these probes also containing peaks in 5hmC or 5mC analyzed (purple and red bars, respectively). From this analysis a small number of the probes were seen to contain peaks of 5hmC (approximately 10%) or 5mC (approximately 2%). Click here for file

Additional file 15 **Figure S13**. Heat map analysis of average 5hmC levels over PPRs in control ('WT') and PB-exposed ('PB') mouse livers. Average 5hmC levels are represented by a color scale ranging from strongly enriched (red, log_2 _+2) to depleted (blue, log_2 _-2). PPRs are clustered by both Euclidian and Ward methods. This analysis reveals that although the majority of PPRs do not reveal strong changes in their 5hmC levels upon PB exposure, certain regions do reveal moderate levels of perturbation. Click here for file

Additional file 16 **Figure S14**. **(a,b) **Scatter plot comparing average promoter changes for 5hmC (a) or 5mC (b) against changes in gene expression levels following 28-day PB exposure. Only a subset of genes show >log_2 _1.5-fold induction (red rectangle) and no correlation with strongly down-regulated genes are noted. Click here for file

Additional file 17 **Figure S15**. Log_2 _fold changes of PB-treated over vehicle (control) samples averages for histone methylation marks (n = 2) and 5mC or hmC (n = 5) for all 20,717 genes. The fold changes for H3K4me2 are calculated in a window of +2/-1 kb of TSS, and gene body for H3K27me3 and H3k36me3 and +1/-1 kb of TSS for 5mC and hmC. The density of genes/promoters is indicated by the grade of blue; data points at the periphery of the main data density are indicated by black dots. The red dot indicates the gene *Cyp2b10*. Click here for file

Additional file 18 **Figure S16**. Log_10 _histone scores of vehicle (control) versus PB treated per mark (group average, n = 2) in 20,717 promoter regions (+2/-1kb of TSS) for H3K4me2 and gene bodies for H3K27me3 and H3K36me3. The dotted green line has intercept 0 and slope 1, the red line is a regression line (R^2^). The further off the diagonal a gene is, the stronger the treatment effect. The density of genes/promoters is indicated by the grade of blue; data points at the periphery of the main data density are indicated by black dots. The red dot indicates the gene *Cyp2b10*. Click here for file

Additional file 19 **Figure S17**. PB-mediated changes to both 5hmC and 5mC levels are not observed over the enhancer elements present on the array. Box plot of 5hmC (purple) and 5mC (red) log2 levels over 1 kb enhancer elements in control or 28-day PB-exposed mouse livers reveal no global changes at these sites. Click here for file

Additional file 20 **Figure S18**. Genes that are unchanged in their expression following PB exposure do not correlate to changes in both DNA and histone modifications. Scatter graph plots display the average changes in epigenetic marks (5hmC, 5mC, H3K4me2, H3K27me3 and H3K36me3) against fold change in expression for 30 genes unchanged in their expression state following 28-day PB treatment. Trend lines are displayed in red with associated Pearson correlation ('cor') and *P*-values. These findings contrast those observed over 30 PB-induced genes (Figure 3b). Click here for file

Additional file 21 **Table S3**. Top 25 genes induced upon 28-day PB exposure. Click here for file

Additional file 22 **Table S4**. Gene Ontology terms for genes induced following PB exposure. Click here for file

Additional file 23 **Figure S19**. Examples of changes to the profiles of histone modifications (ChIP-Seq) and DNA modification changes (MeDIP/HmeDIP-chip) across select *Cyp2b *and *Gst *genes. Patterns of log_2 _changes in the signals of 5mC (red), 5hmC (purple), H3K36me3 (green), H3K27me3 (orange) and H3K4me2 (blue) are plotted over these regions. The Y-axis of ChIP-Seq samples were plotted on a scale of +70 to -70+ reads whilst promoter arrays (5hmC and 5mC) plotted from +1.5 log_2 _to -1.5 log_2 _values. Click here for file

Additional file 24 **Figure S20**. Plots of average changes in epigenetic marks over *Gst *gene family in comparison to genes unaffected in their gene expression states following 28-day PB exposure. Average changes in the log_2 _scores (DNA modifications) or fold change in the number of reads (histone modifications) of the marks are plotted against regions outlined earlier in Figure 1. Error bars are standard error and points showing significant deviation from unaffected genes (Willcox test, *P*-value < 0.005) are denoted by the red asterisk. Red dotted line represents 0 change in epigenetic mark upon PB exposure. Click here for file

Additional file 25 **Table S5**. List of DNA primers used in this study. Click here for file
