# Supplementary figures and images for: Non-genotoxic carcinogen exposure induces defined changes in the 5-hydroxymethylome
Source: Genome Biol. 2012 Oct 3;13(10):R93. doi: 10.1186/gb-2012-13-10-r93 (PMC3491421; doi:10.1186/gb-2012-13-10-r93)

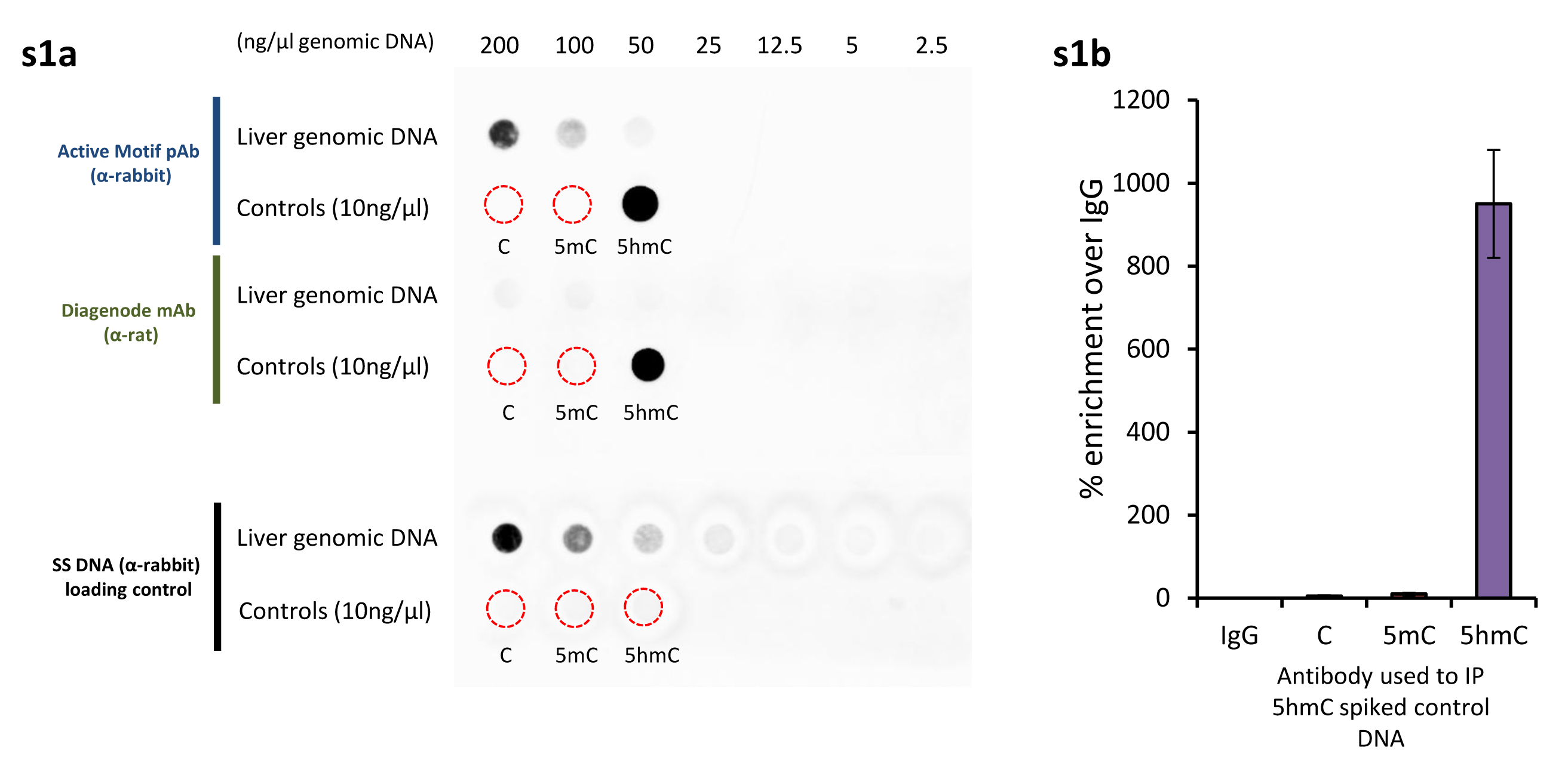

Supplement: Additional file 1 — Figure S1. (a) Dot blot comparing the specificities and sensitivities of two commercially available 5hmC antibodies. Antibodies were tested over a dilution gradient of genomic liver DNA (200 ng/μl to 2.5 ng/μl) as well as for their relative affinities against PCR products synthesized in the presence of either dCTP, dmCTP or d5hmCTP [68]. Although both antibodies can distinguish 5hmC from 5mC and non-modified C (see controls), only the polyclonal (pAB) α-rabbit antibody (Active motif) can detect 5hmC in genomic mouse liver DNA. It must be noted that the relative levels of 5hmC present in the control PCRs are far greater than that in the genomic DNA. (b) The HmeDIP technique is highly specific for 5hmC-modified DNA relative to unmodified or methylated DNA. qPCR was run on immunoprecipitated material that had been spiked with a 5hmC-rich DNA sequence. Enrichment of the 5hmC rich control DNA was only observed when immunoprecipitated with the 5hmC antibody and not with an antibodies against 5mC, non-modified C or IgG. [file gb-2012-13-10-r93-S1.tiff]

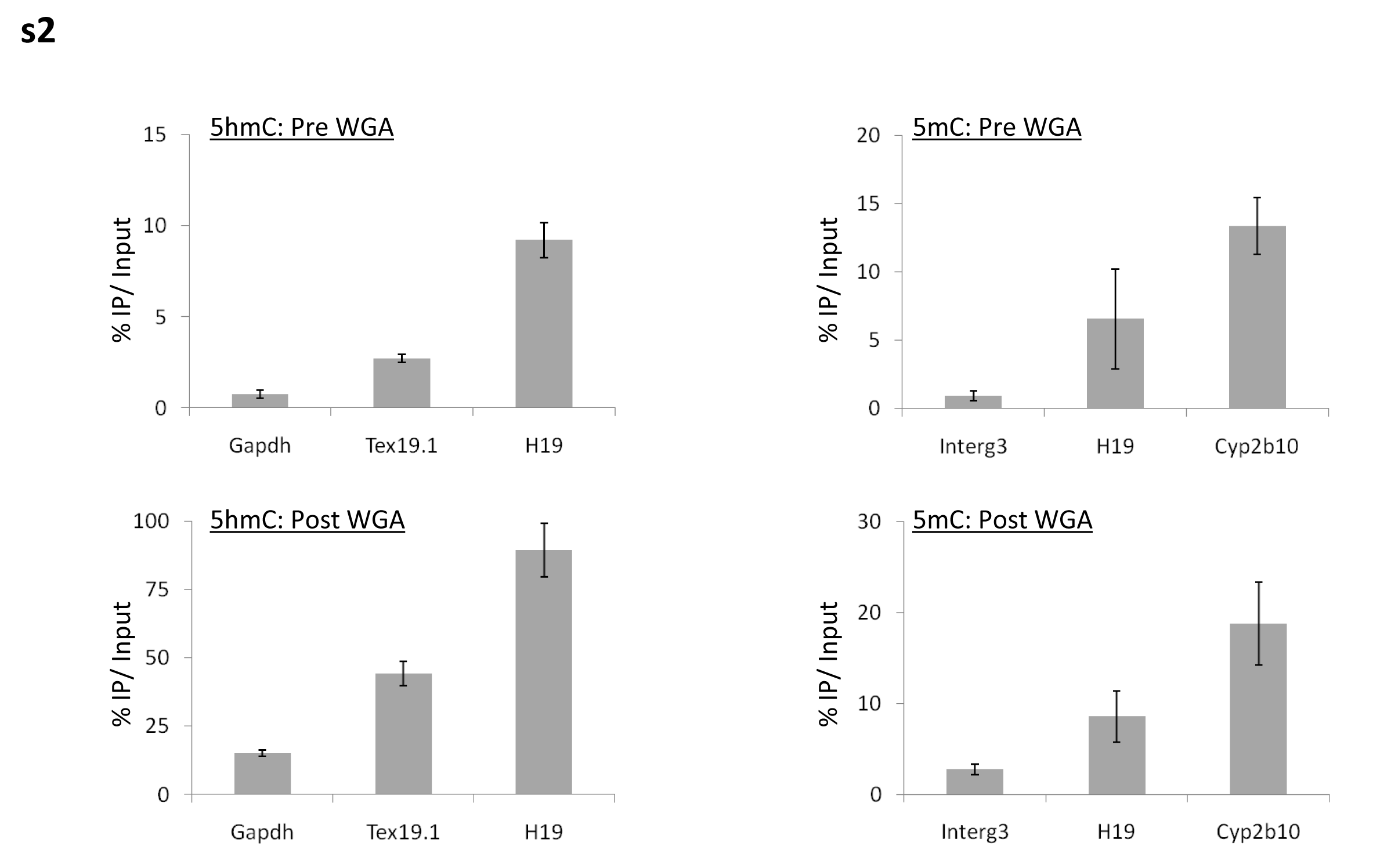

Supplement: Additional file 2 — Figure S2. qPCR analysis on pre- and post-whole genome amplification (WGA) material introduced no bias upon amplification. Confirmatory qPCR showing hmC enrichment at Tex19.1 and H19 promoter regions (known hmC-enriched loci) alongside a 5hmC-negative control region (Gapdh promoter) both prior to and after WGA. 5mC enrichment was tested in a similar way using positive control regions of the H19 ICR and Cyp2b10 promoter regions and a negative ICR. [file gb-2012-13-10-r93-S2.tiff]

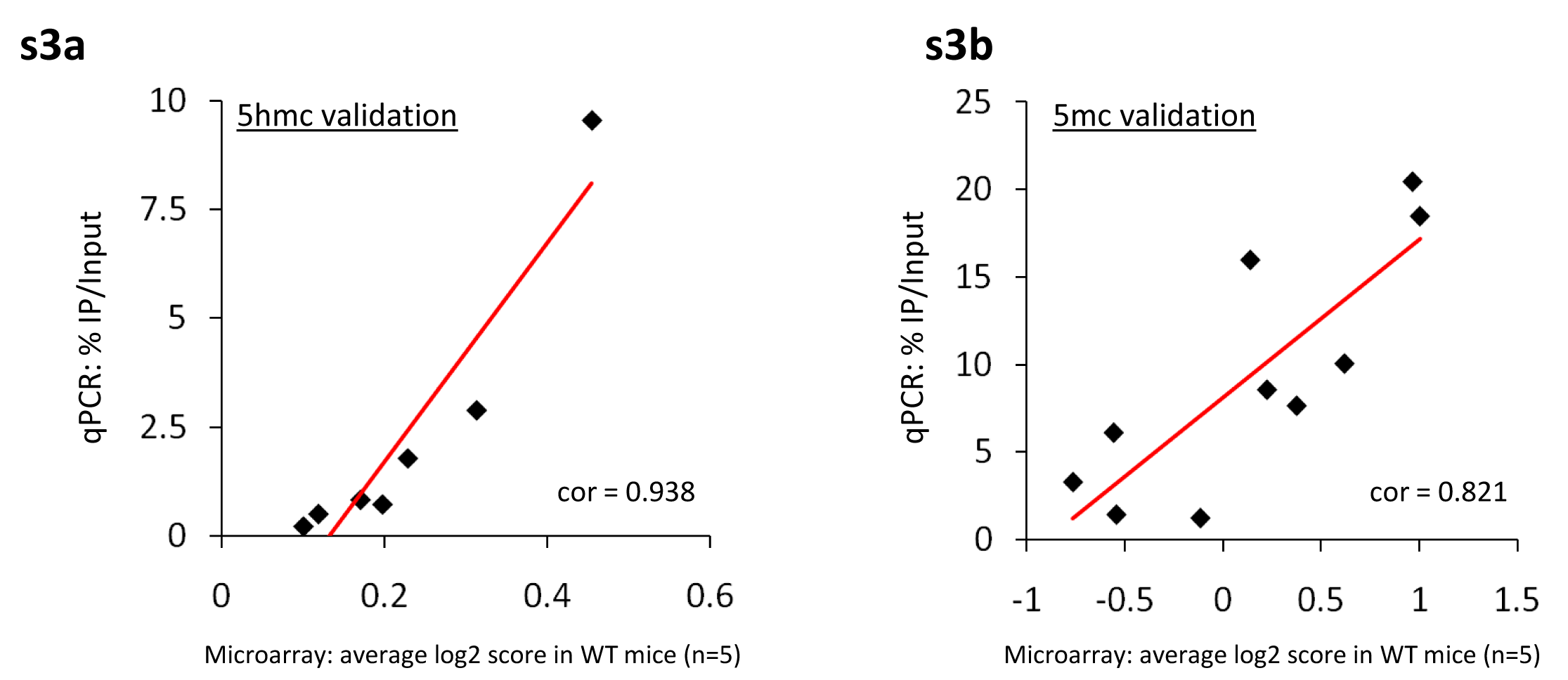

Supplement: Additional file 3 — Figure S3. (a,b) Validation of microarray profiling by qPCR. Correlation between HmeDIP and MeDIP qPCR enrichment values (% IP/input) to log2 values seen on the promoter microarrays is presented for 5hmC (a) and 5mC (b). Trend line (red) and R2 values are also shown. Pearson correlation values ('cor') shown alongside represent the closeness of fit between qPCR enrichment values (Y-axis) and log2 scores taken from microarray data (X-axis). Loci used for validation of HmeDIP were as follows: upstream region, 5' and 3' promoter regions of Cyp2b10, promoter regions of Tex19.1, Actb, Gapdh and H19. Loci used for the validation of MeDIP are as follows: ICRs of H19 and Gnas, intron 1 of Cyp2b10 and promoter regions of CSA, Pou3f4, Ccdc34, Tyms, Ccr1, Cbx2 and Tacr3 (see primer list in Additional file 25). [file gb-2012-13-10-r93-S3.tiff]

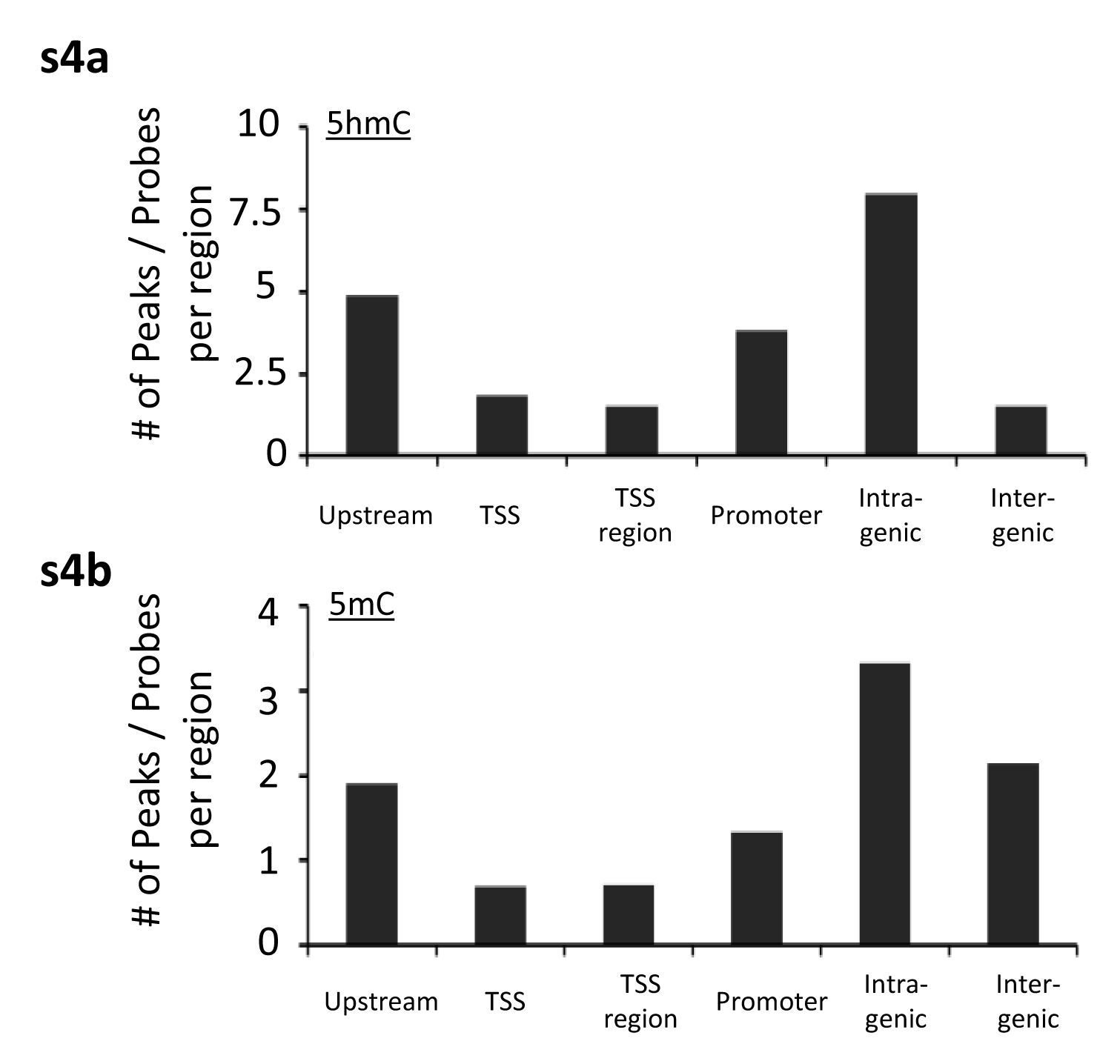

Supplement: Additional file 4 — Figure S4. Normalized peak finding data for 5hmC and 5mC based on data from Figure 1c. The number of peaks represented in Figure 1c for both 5hmC and 5mC were divided by the number of probes covering each region (Figure 1a,c) to remove coverage bias. (a,b) Overall there are no large changes in the distribution of 5hmC (a) and 5mC (b) enriched peaks upon normalization with the exception of an increase in upstream 5hmC and 5mC peaks and reduction of inter-genic 5hmC peaks. [file gb-2012-13-10-r93-S4.tiff]

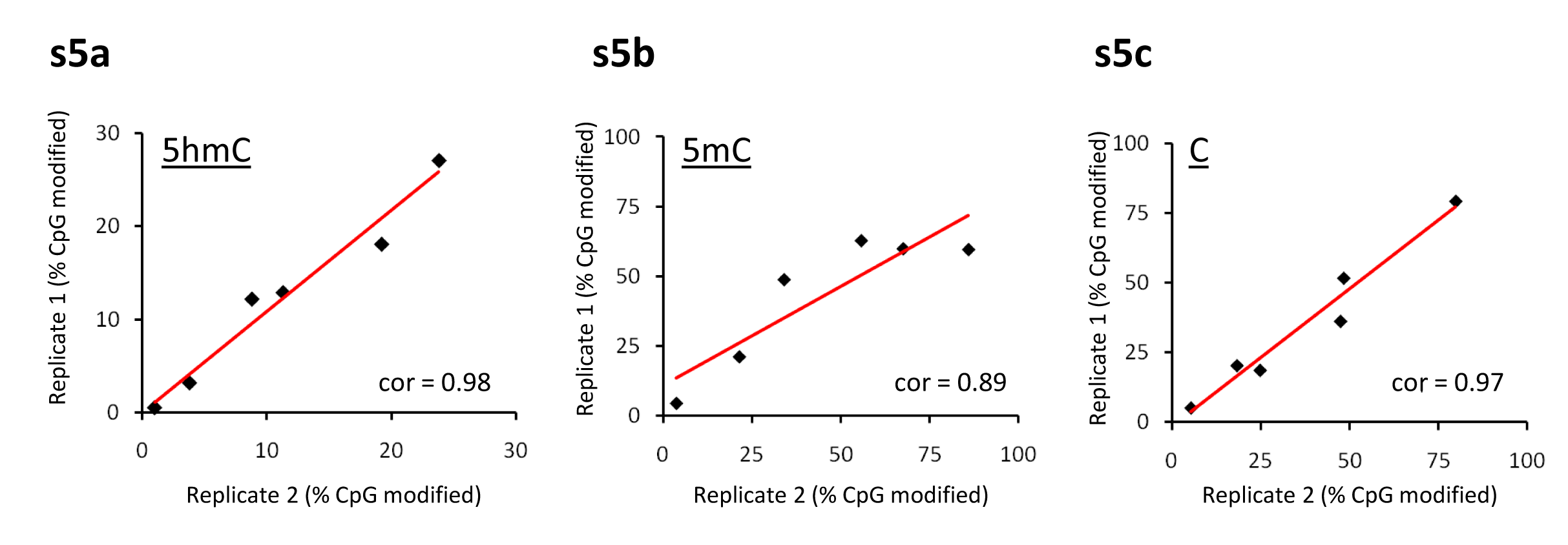

Supplement: Additional file 5 — Figure S5. Validation of 'EpiMark' quantitative analysis techniques reveals highly similar levels of the DNA modifications between individuals. (a-c) The values of 5hmCpG (a), 5mCpG (b) and CpG (c)were plotted between two biological replicates to test the reproducibility of the result. Loci tested were as follows: TSS regions of Gapdh, H19 and Tspan10, an intra-genic region of Gstm3, a region upstream of the Cyp2b10 promoter and an inter-genic region spanning chr7: 149709621-149709863 (see primer list in Additional file 25). All modified forms of CpG are reproducible between individuals over the six loci. Pearson correlation values ('cor') are shown alongside representing the reproducibility of results. [file gb-2012-13-10-r93-S5.tiff]

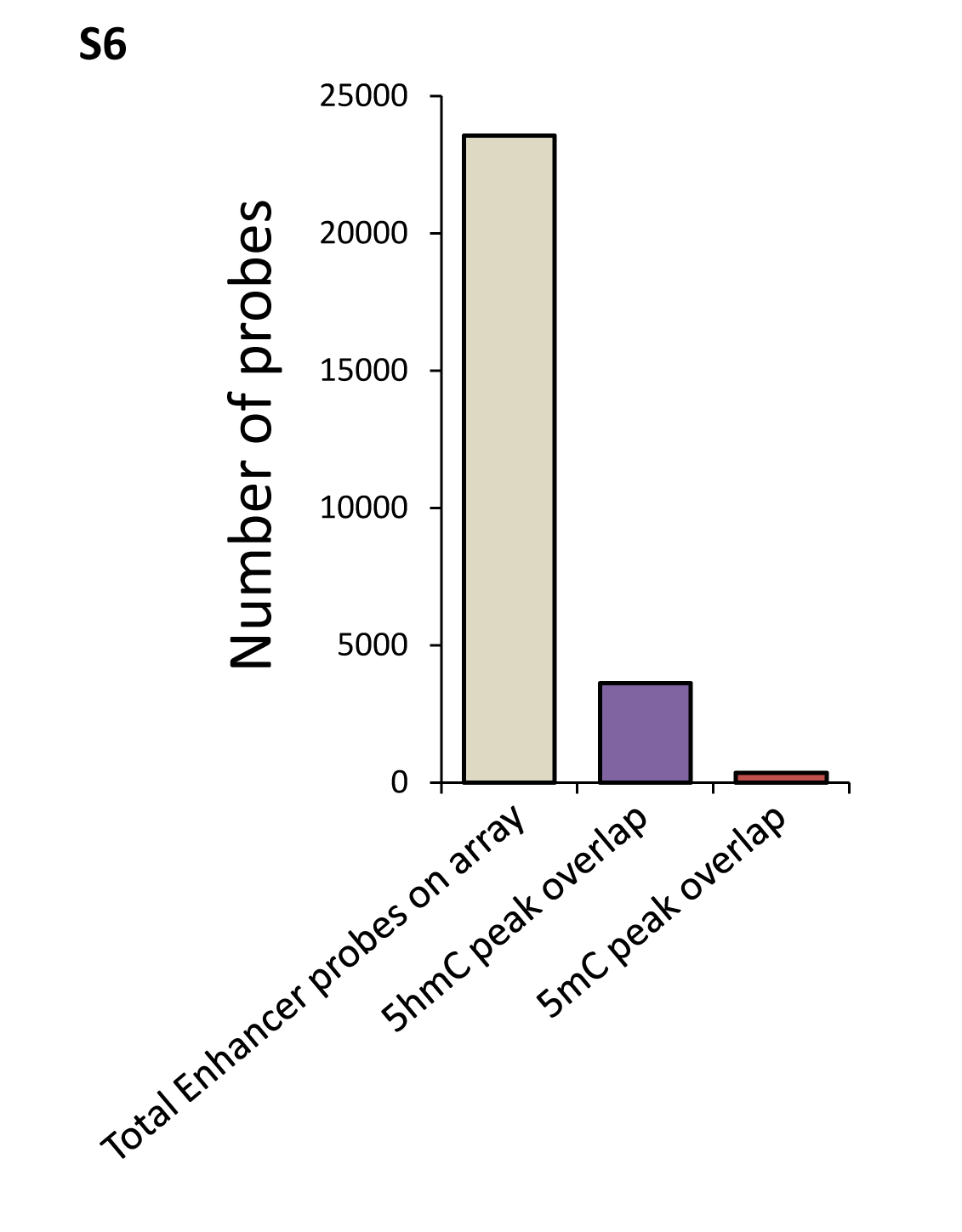

Supplement: Additional file 6 — Figure S6. A subset of enhancer elements present on the promoter array overlap with peaks of 5hmC. Total number of probes that overlap with 1 kb long enhancer elements defined by Shen et al. [75] (grey bar) are plotted next to enhancer probes that also contain a peak of either 5hmC (purple bar) or 5mC (red bar). Overall, approximately 15% of enhancer probes on the array overlap with peaks of 5hmC. [file gb-2012-13-10-r93-S6.tiff]

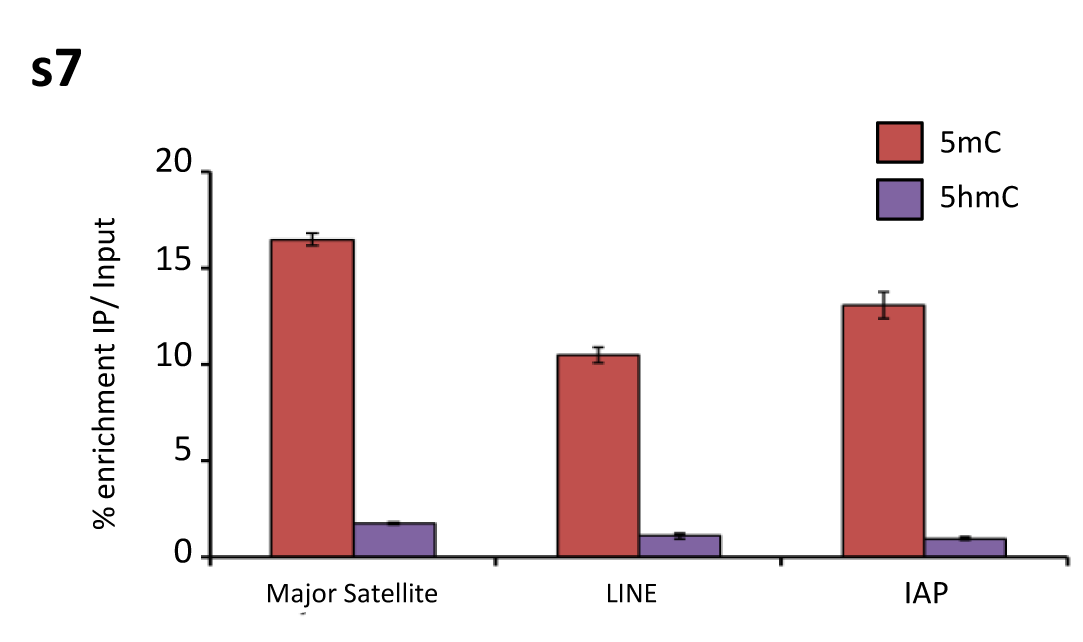

Supplement: Additional file 7 — Figure S7. qPCR analysis of 5hmC (purple) and 5mC (red) levels at repeat elements (major satellites, LINEs and IAPs) in liver DNA. Enrichment values are plotted as percentage IP relative to the input. [file gb-2012-13-10-r93-S7.tiff]

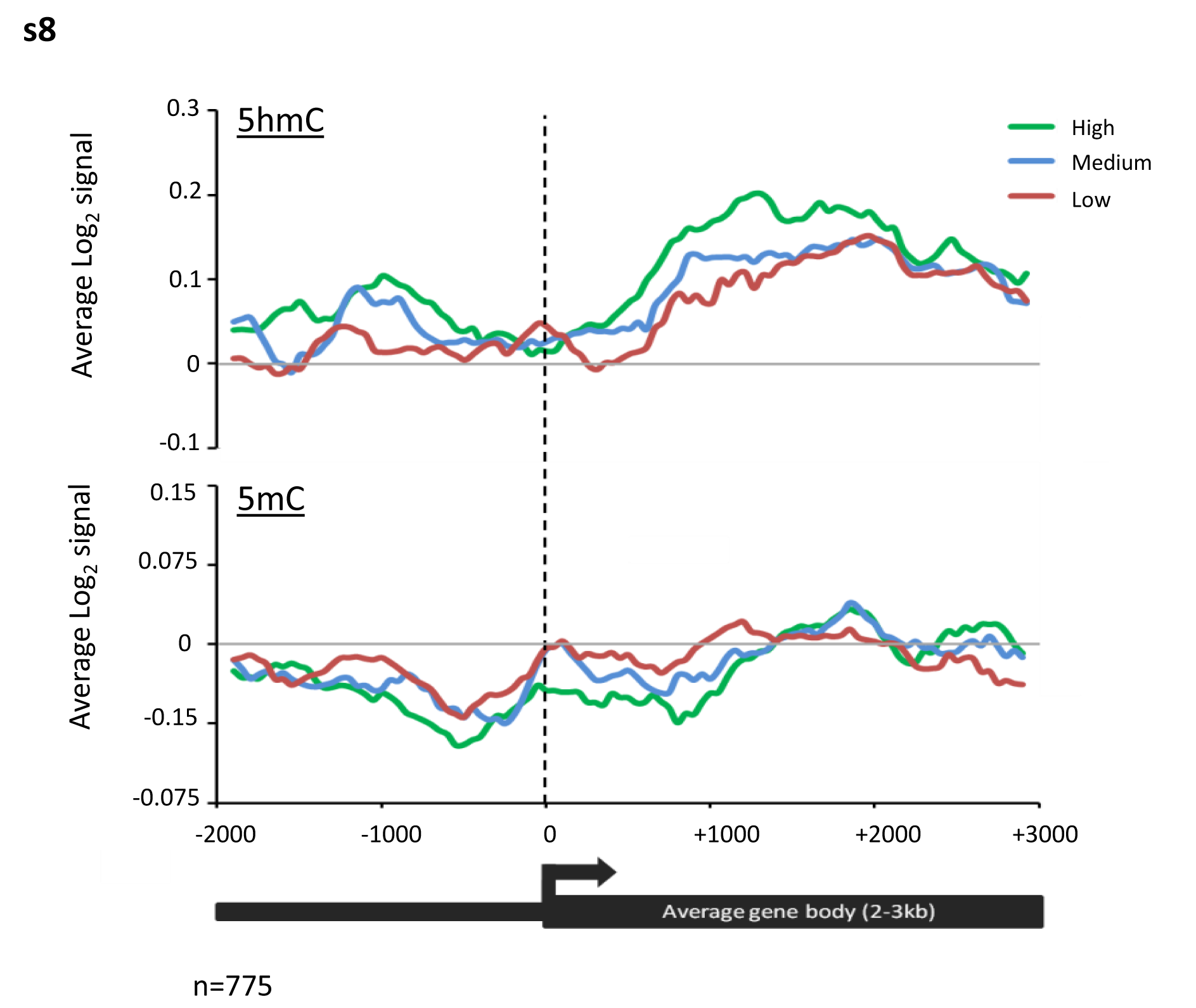

Supplement: Additional file 8 — Figure S8. Sliding window analysis of 5hmC and 5mC over full-length genes (n = 775) covered on the array. Levels of 5hmC increase throughout the body of a gene in a transcription-dependant manner. Expression levels were calculated within the 775 'small' gene set. Top 25% = highly transcribed (green), bottom 25% = lowly transcribed (red). In contrast to 5hmC, levels of 5mC are higher over the TSS of lowly expressed genes and show less of a correlation with transcriptional levels throughout the gene body. [file gb-2012-13-10-r93-S8.tiff]

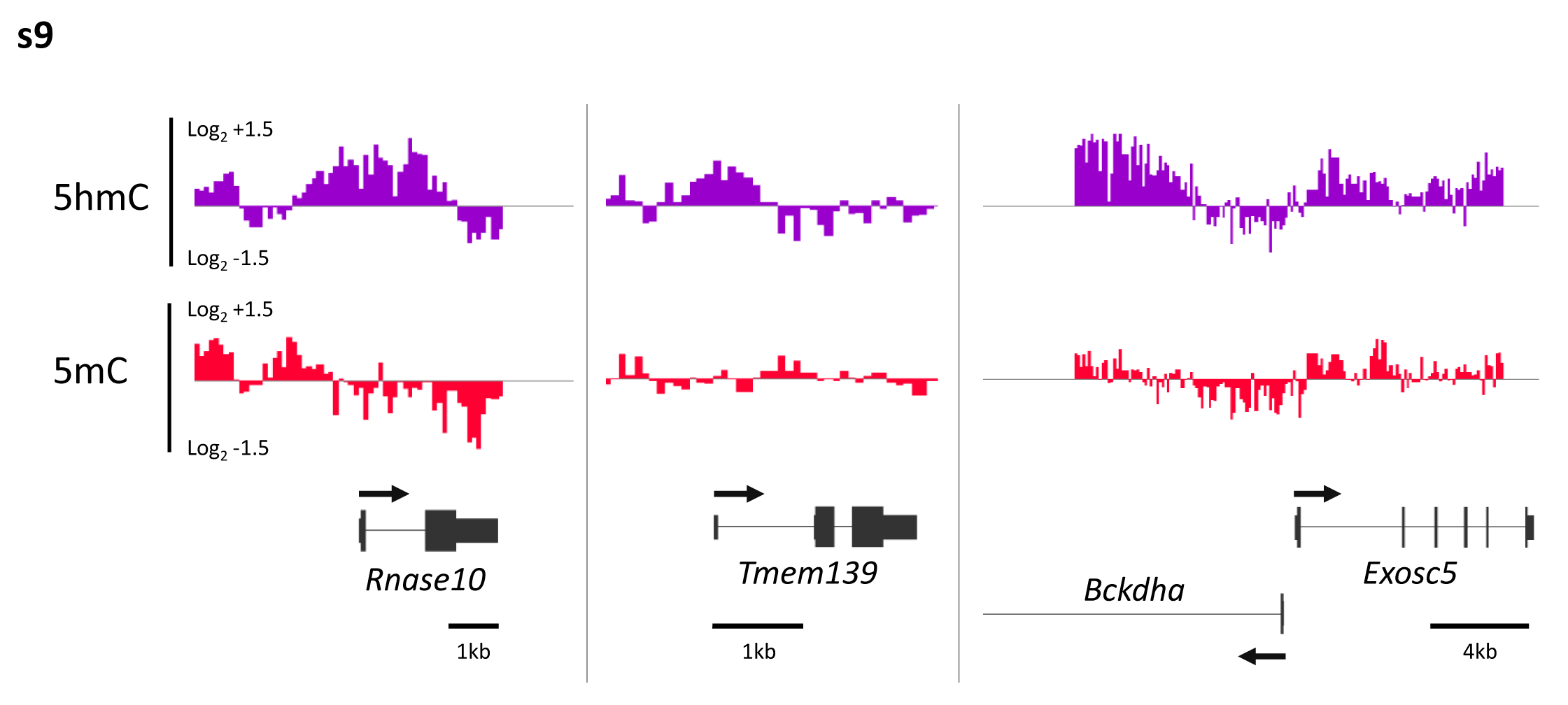

Supplement: Additional file 9 — Figure S9. Examples of 5hmC-enriched TSS regions along with more typical 5hmC-depleted TSS regions. Two examples of 5hmC-enriched genes are shown on the left (Rnas10 and Tmem139). In comparison, a more typical (5hmC depleted) TSS region is shown on the right for Bckdha and Exosc5. All scales on the Y-axis are from log2 1.5 to -log2 1.5. Individual X-axis scale bars are represented by a black bar. The 5hmC profile is in purple and 5mC in red; the transcription start site (black arrow) and gene structure are also illustrated. Exons are shown as perpendicular lines. [file gb-2012-13-10-r93-S9.tiff]

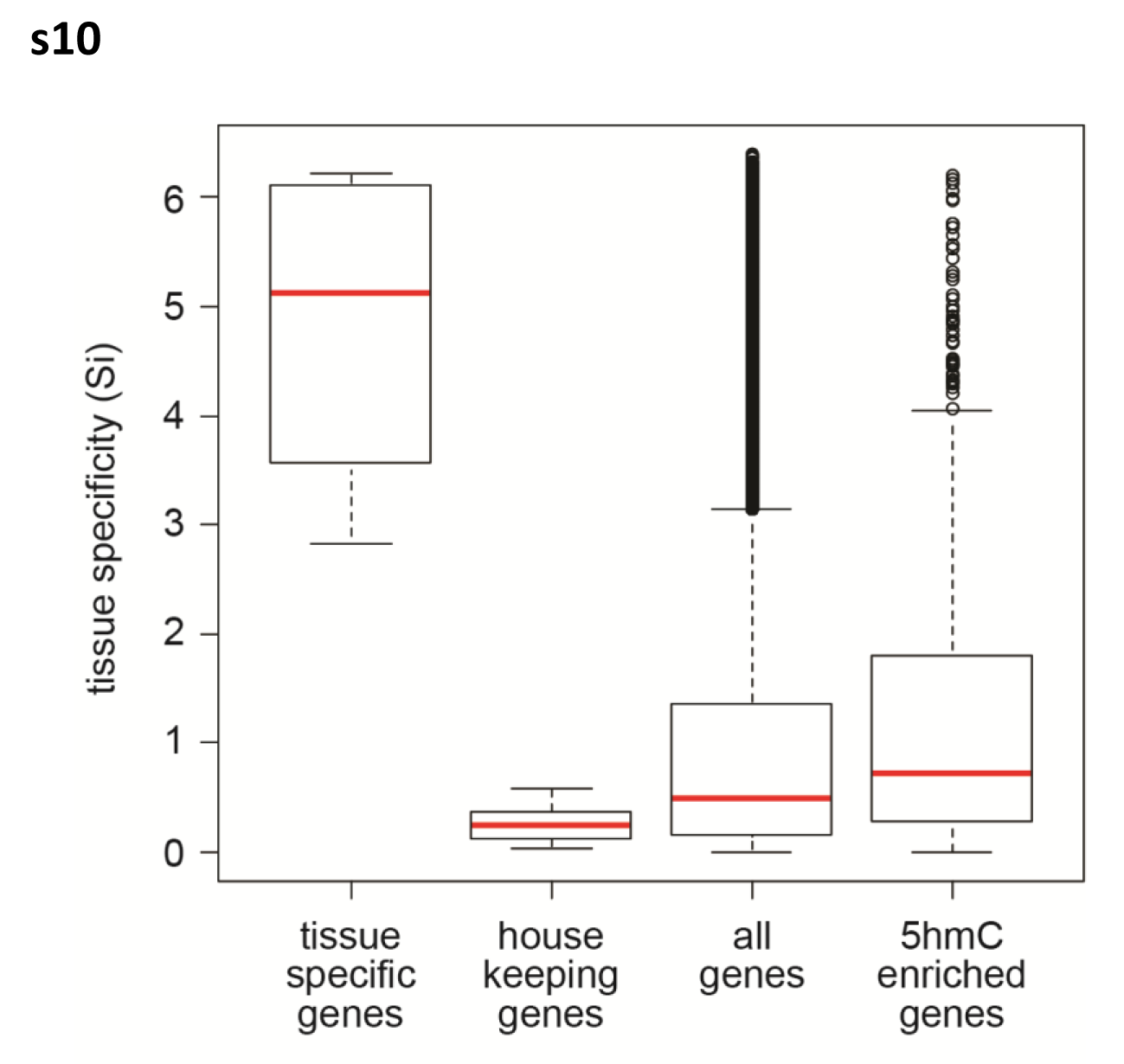

Supplement: Additional file 12 — Figure S10. Genes with 5hmC enrichment over their TSS show some enrichment towards those that are expressed in a tissue-specific manner. The relative levels of tissue specificity were plotted (see Materials and methods; Si value of 0 = non tissue specific, Si value of 6.2 = highly tissue specific) against groups of genes with high tissue specificity as well as housekeeping genes. In comparison to all of the genes on the array there is a small but significant (P-value < 0.001) increase in the level of tissue specificity for genes with 5hmC-enriched regions spanning the TSS. The red lines represent the median score of the group of genes. The upper circles represent outliers of the majority of genes. [file gb-2012-13-10-r93-S12.tiff]

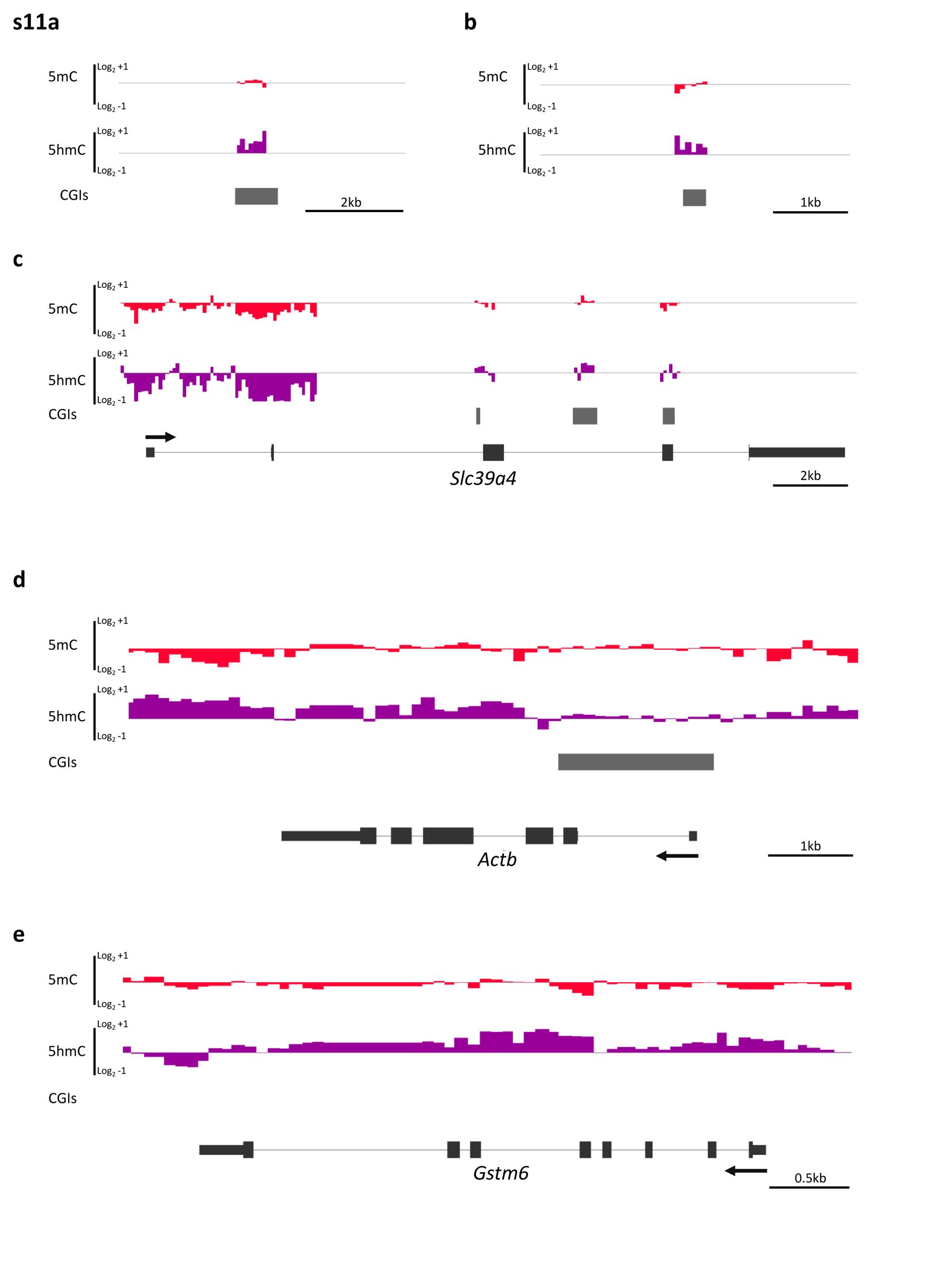

Supplement: Additional file 13 — Figure S11. Examples of CGIs with high CpG density (HCP) that are enriched for 5hmC. Although the majority of probes covering CGIs do not contain peaks of 5hmC, those that do show a striking enrichment over certain loci. (a-c) The majority of the 5hmC-enriched probes mapping to CGIs are inter-genic (a,b) or intra-genic (c). Intragenic CGIs in (a) are found at chr9:101,153,320-101,154,414 (left panel) and chr11:90,279,237-90,279,648 (right panel). Intergenic CGIs shown in the example in (c) are found within the gene Zip4. (d,e) Promoters with CGIs (Actb is the example (d)) are also found to be largely depleted in the 5hmC modification in contrast to those that lack CGIs (Gstm6 is the example (e)). All scales on the Y-axis are from log2 +2 to -log2 2. Transcription start site (black arrow) and gene structure are illustrated below each example. Exons are shown as perpendicular lines. Scale bars are represented at the bottom right of each figure. [file gb-2012-13-10-r93-S13.tiff]

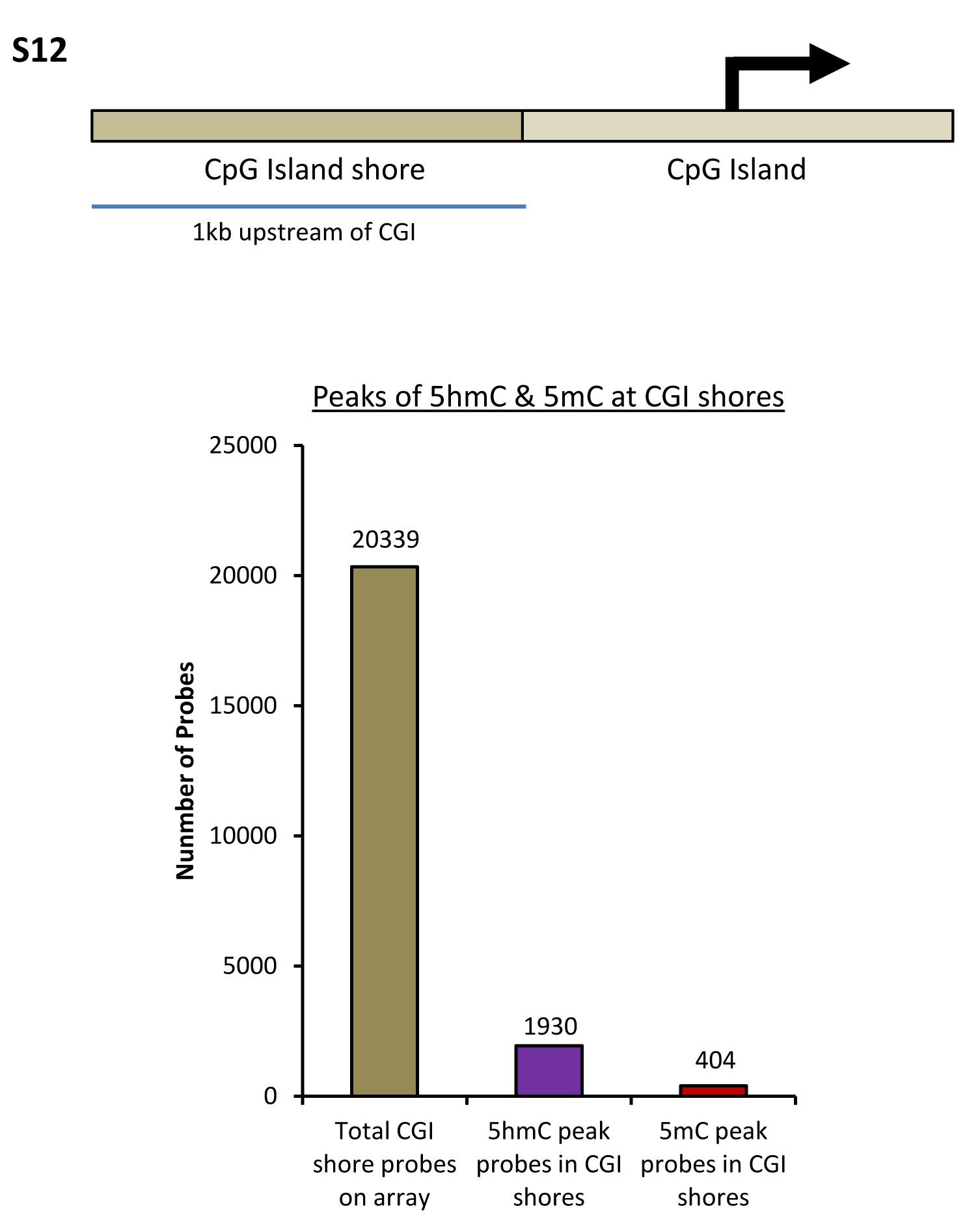

Supplement: Additional file 14 — Figure S12. 5hmC and 5mC levels are not enriched over 'CGI shores'. The total number of probes found over regions 1 kb upstream of annotated CGIs were selected (upper panel and lower panel grey bar) and the number of these probes also containing peaks in 5hmC or 5mC analyzed (purple and red bars, respectively). From this analysis a small number of the probes were seen to contain peaks of 5hmC (approximately 10%) or 5mC (approximately 2%). [file gb-2012-13-10-r93-S14.tiff]

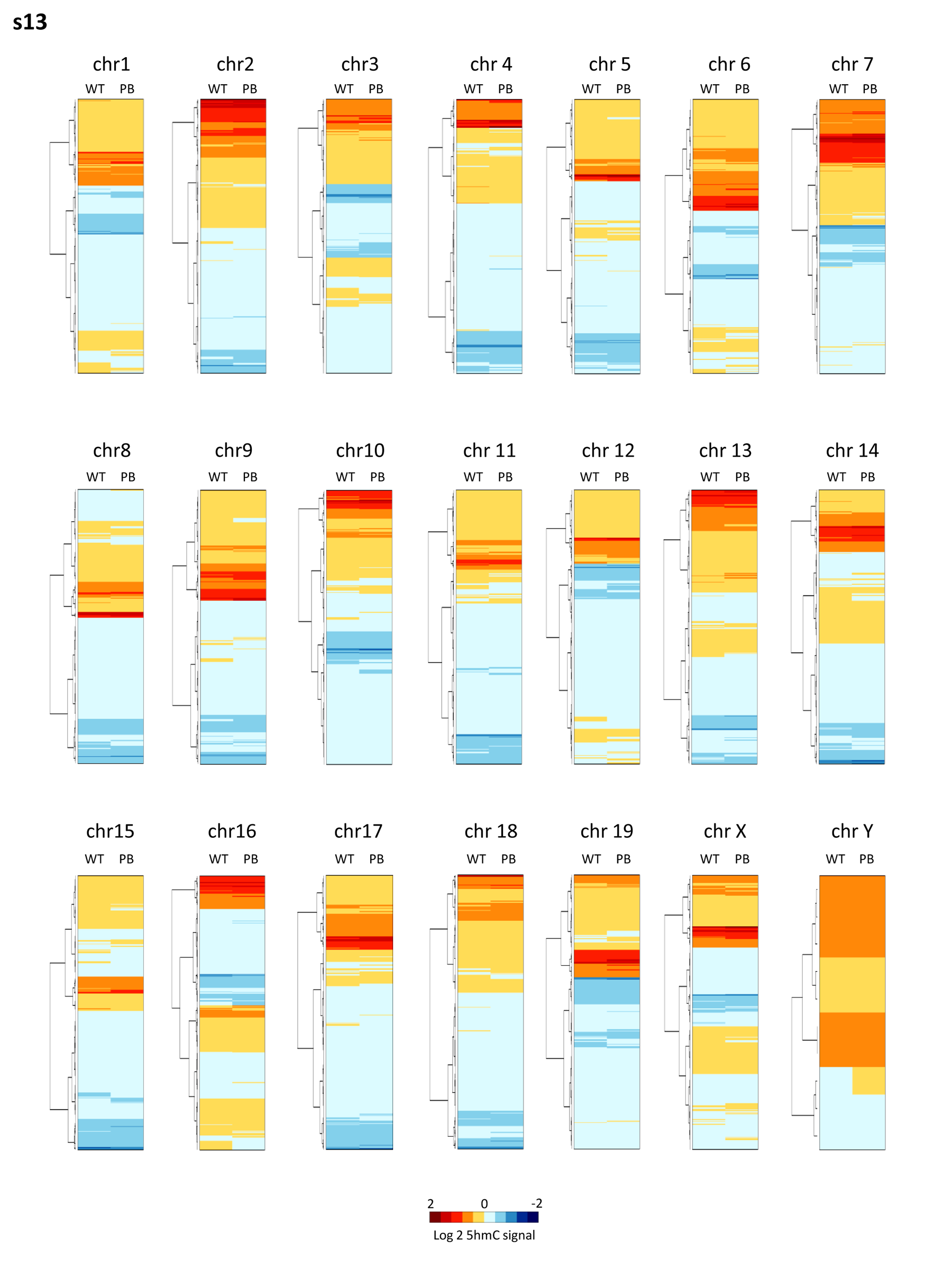

Supplement: Additional file 15 — Figure S13. Heat map analysis of average 5hmC levels over PPRs in control ('WT') and PB-exposed ('PB') mouse livers. Average 5hmC levels are represented by a color scale ranging from strongly enriched (red, log2 +2) to depleted (blue, log2 -2). PPRs are clustered by both Euclidian and Ward methods. This analysis reveals that although the majority of PPRs do not reveal strong changes in their 5hmC levels upon PB exposure, certain regions do reveal moderate levels of perturbation. [file gb-2012-13-10-r93-S15.tiff]

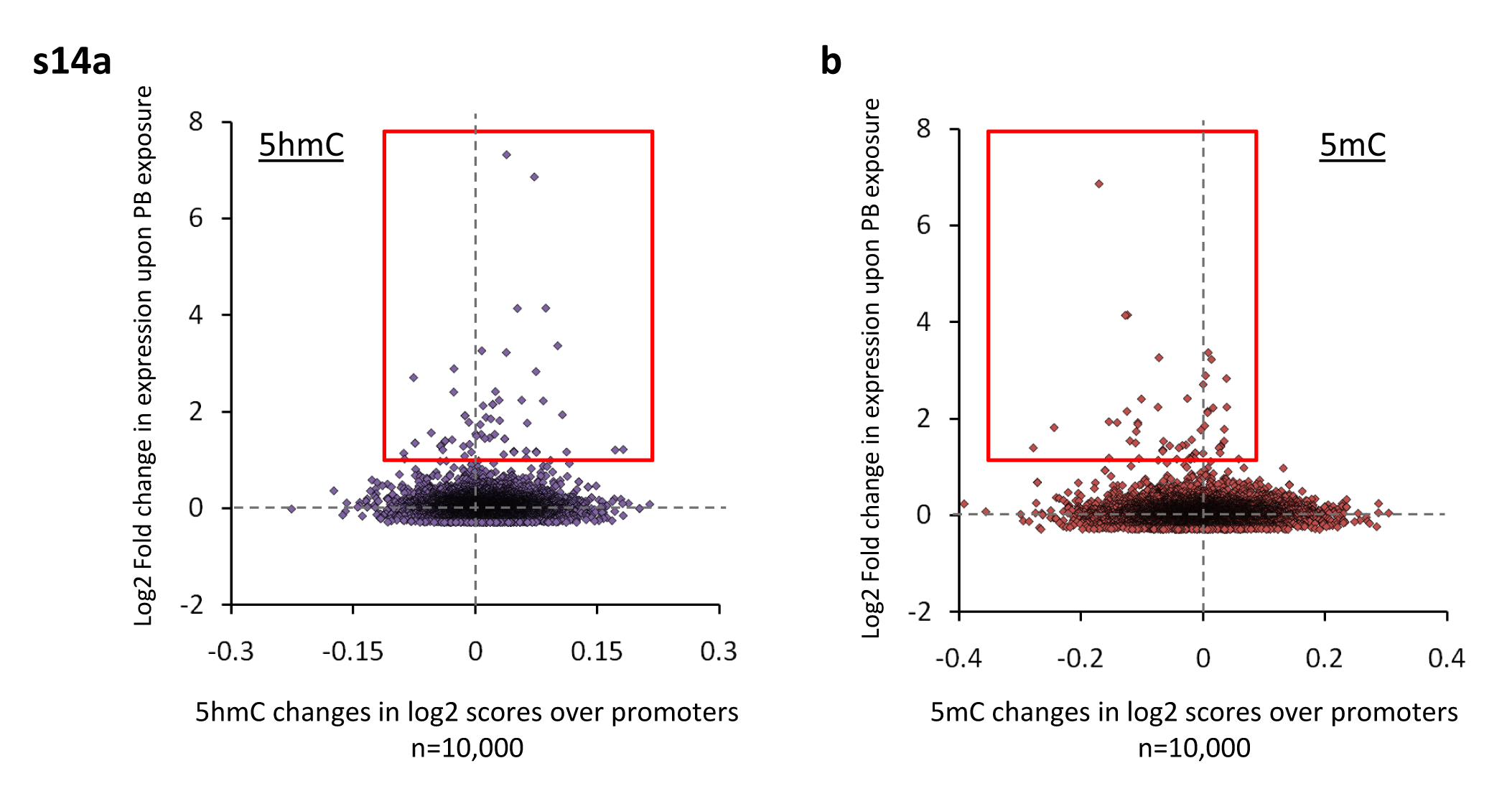

Supplement: Additional file 16 — Figure S14. (a,b) Scatter plot comparing average promoter changes for 5hmC (a) or 5mC (b) against changes in gene expression levels following 28-day PB exposure. Only a subset of genes show >log2 1.5-fold induction (red rectangle) and no correlation with strongly down-regulated genes are noted. [file gb-2012-13-10-r93-S16.tiff]

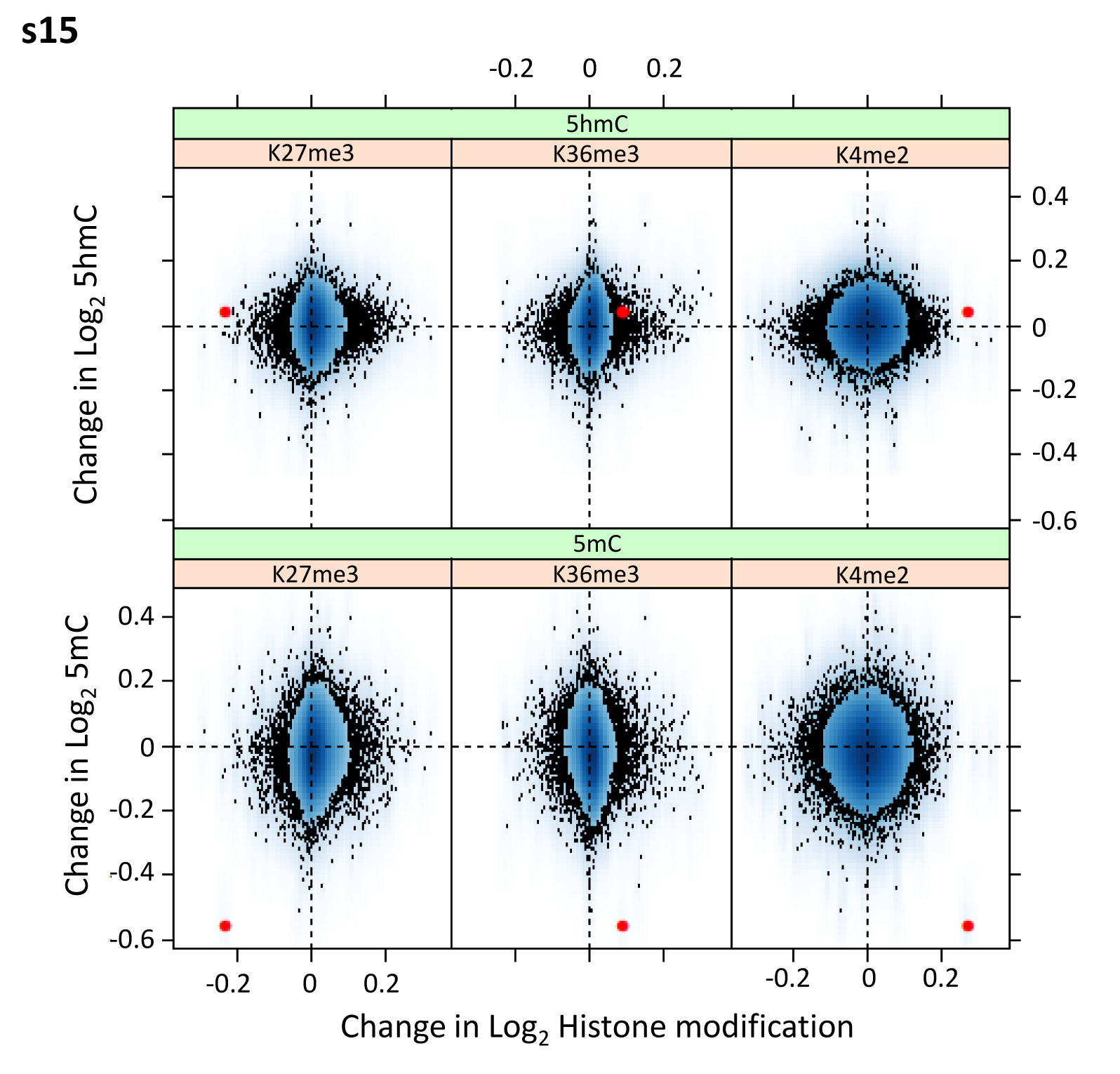

Supplement: Additional file 17 — Figure S15. Log2 fold changes of PB-treated over vehicle (control) samples averages for histone methylation marks (n = 2) and 5mC or hmC (n = 5) for all 20,717 genes. The fold changes for H3K4me2 are calculated in a window of +2/-1 kb of TSS, and gene body for H3K27me3 and H3k36me3 and +1/-1 kb of TSS for 5mC and hmC. The density of genes/promoters is indicated by the grade of blue; data points at the periphery of the main data density are indicated by black dots. The red dot indicates the gene Cyp2b10. [file gb-2012-13-10-r93-S17.tiff]

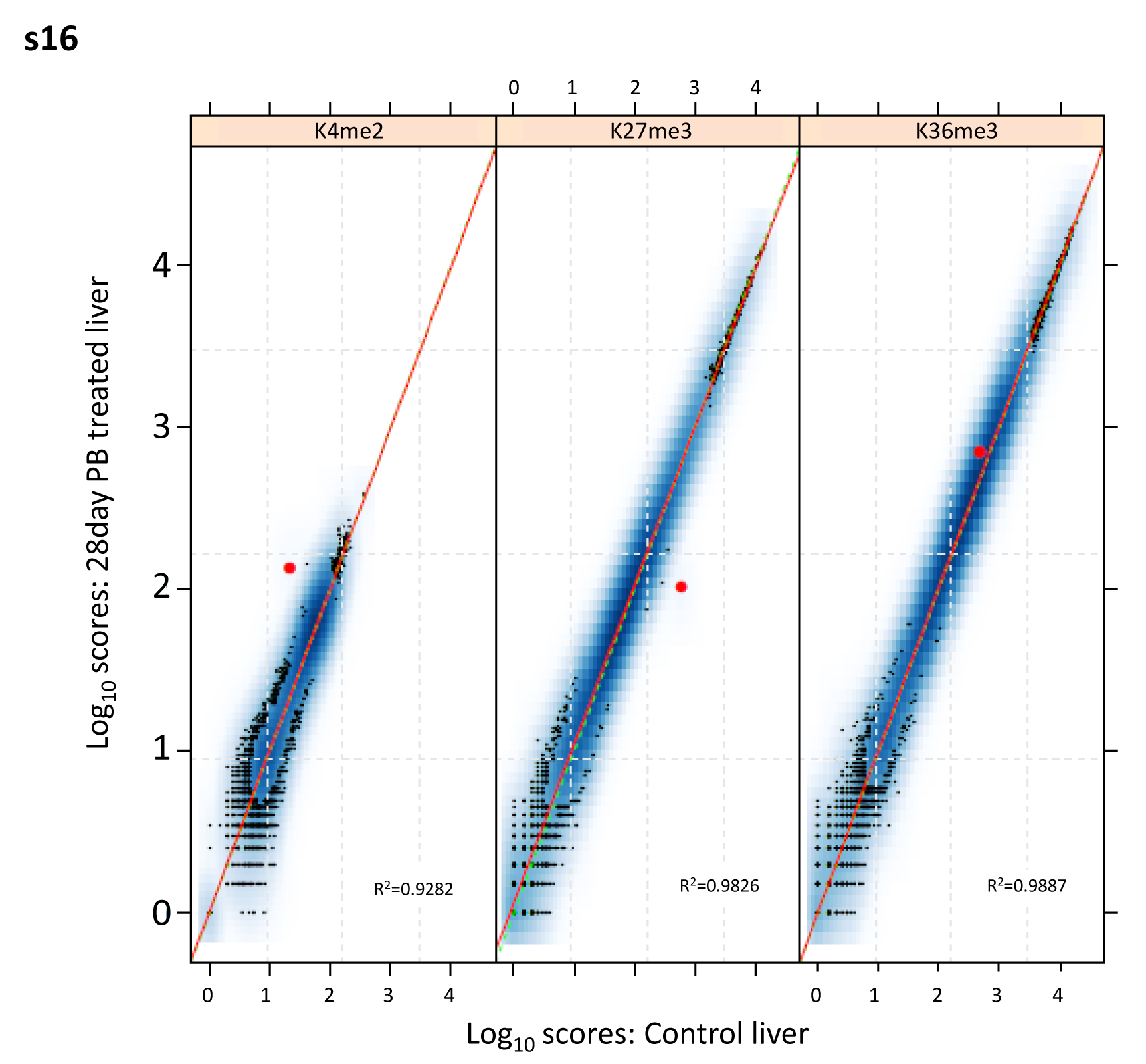

Supplement: Additional file 18 — Figure S16. Log10 histone scores of vehicle (control) versus PB treated per mark (group average, n = 2) in 20,717 promoter regions (+2/-1kb of TSS) for H3K4me2 and gene bodies for H3K27me3 and H3K36me3. The dotted green line has intercept 0 and slope 1, the red line is a regression line (R2). The further off the diagonal a gene is, the stronger the treatment effect. The density of genes/promoters is indicated by the grade of blue; data points at the periphery of the main data density are indicated by black dots. The red dot indicates the gene Cyp2b10. [file gb-2012-13-10-r93-S18.tiff]

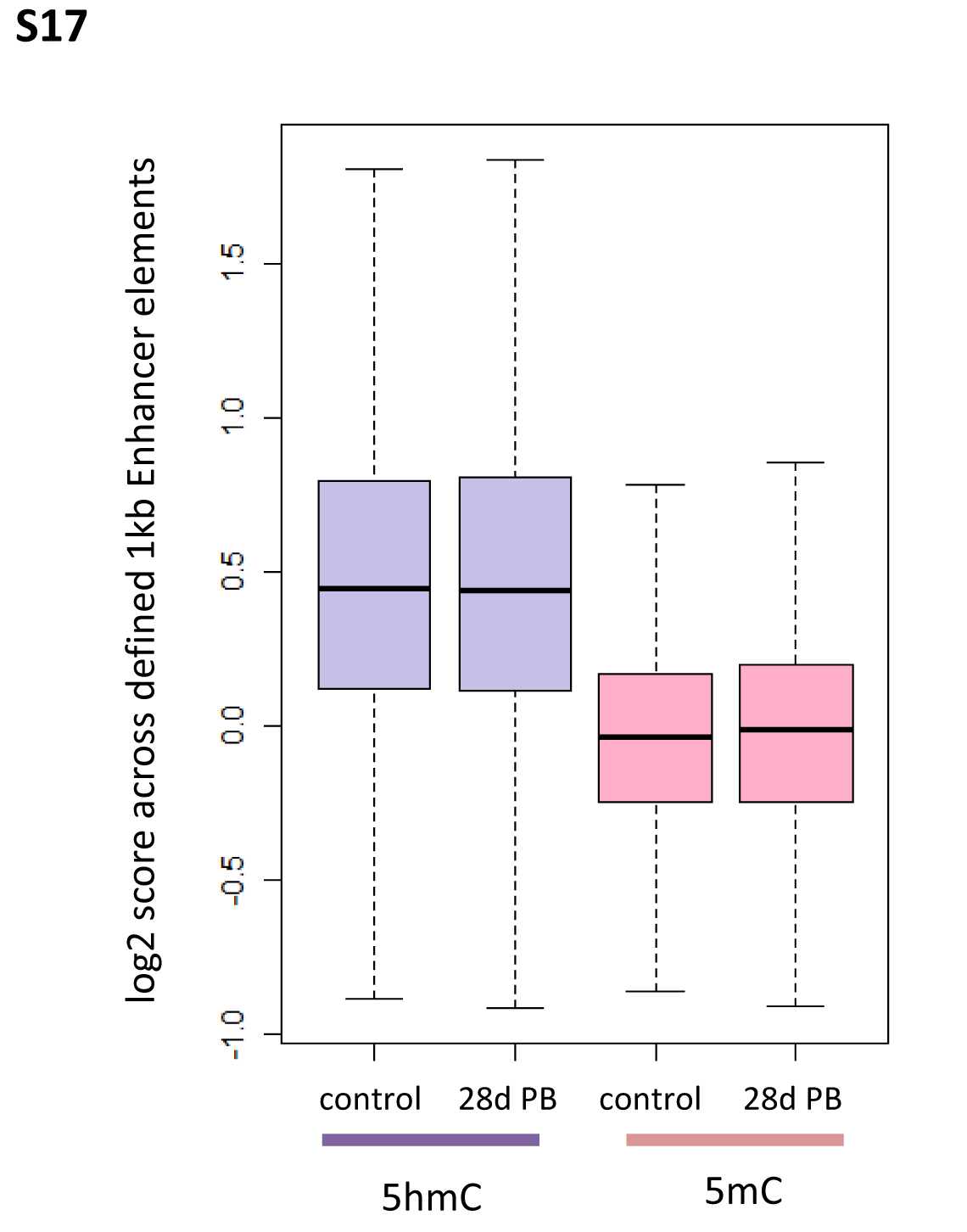

Supplement: Additional file 19 — Figure S17. PB-mediated changes to both 5hmC and 5mC levels are not observed over the enhancer elements present on the array. Box plot of 5hmC (purple) and 5mC (red) log2 levels over 1 kb enhancer elements in control or 28-day PB-exposed mouse livers reveal no global changes at these sites. [file gb-2012-13-10-r93-S19.tiff]

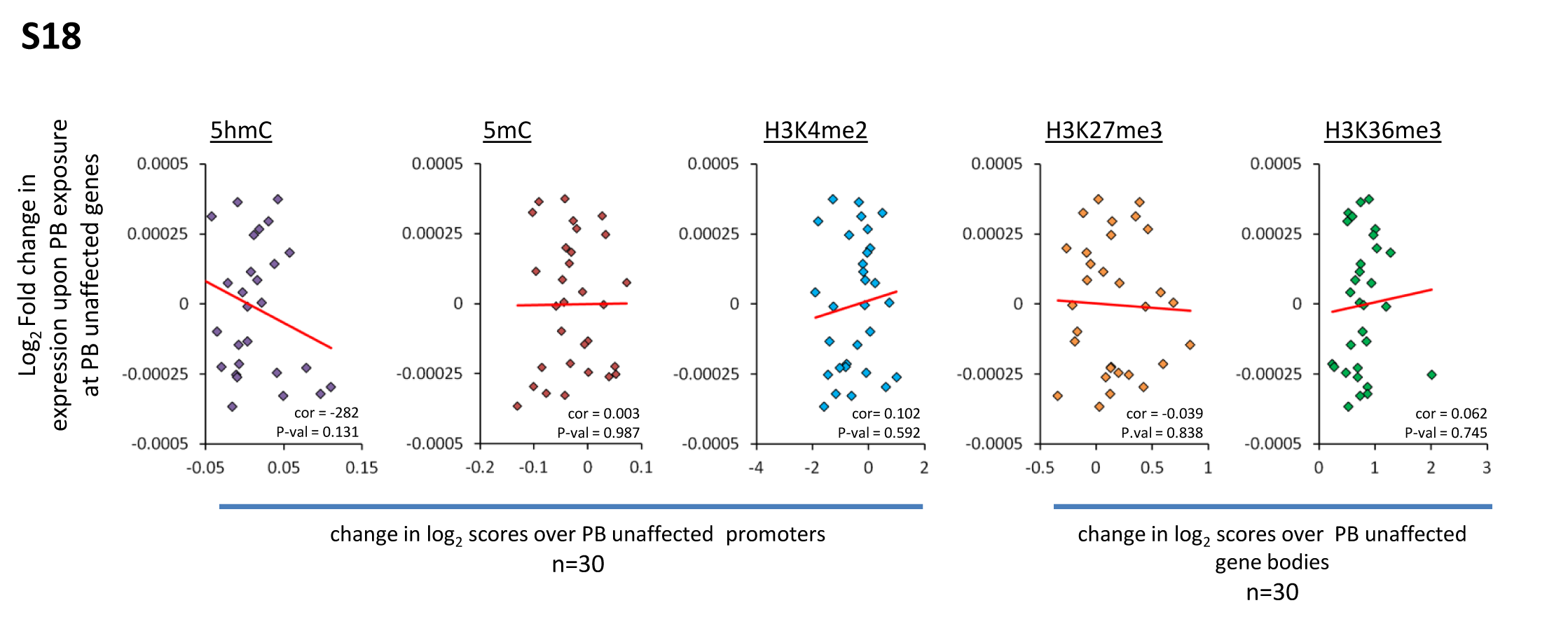

Supplement: Additional file 20 — Figure S18. Genes that are unchanged in their expression following PB exposure do not correlate to changes in both DNA and histone modifications. Scatter graph plots display the average changes in epigenetic marks (5hmC, 5mC, H3K4me2, H3K27me3 and H3K36me3) against fold change in expression for 30 genes unchanged in their expression state following 28-day PB treatment. Trend lines are displayed in red with associated Pearson correlation ('cor') and P-values. These findings contrast those observed over 30 PB-induced genes (Figure 3b). [file gb-2012-13-10-r93-S20.tiff]

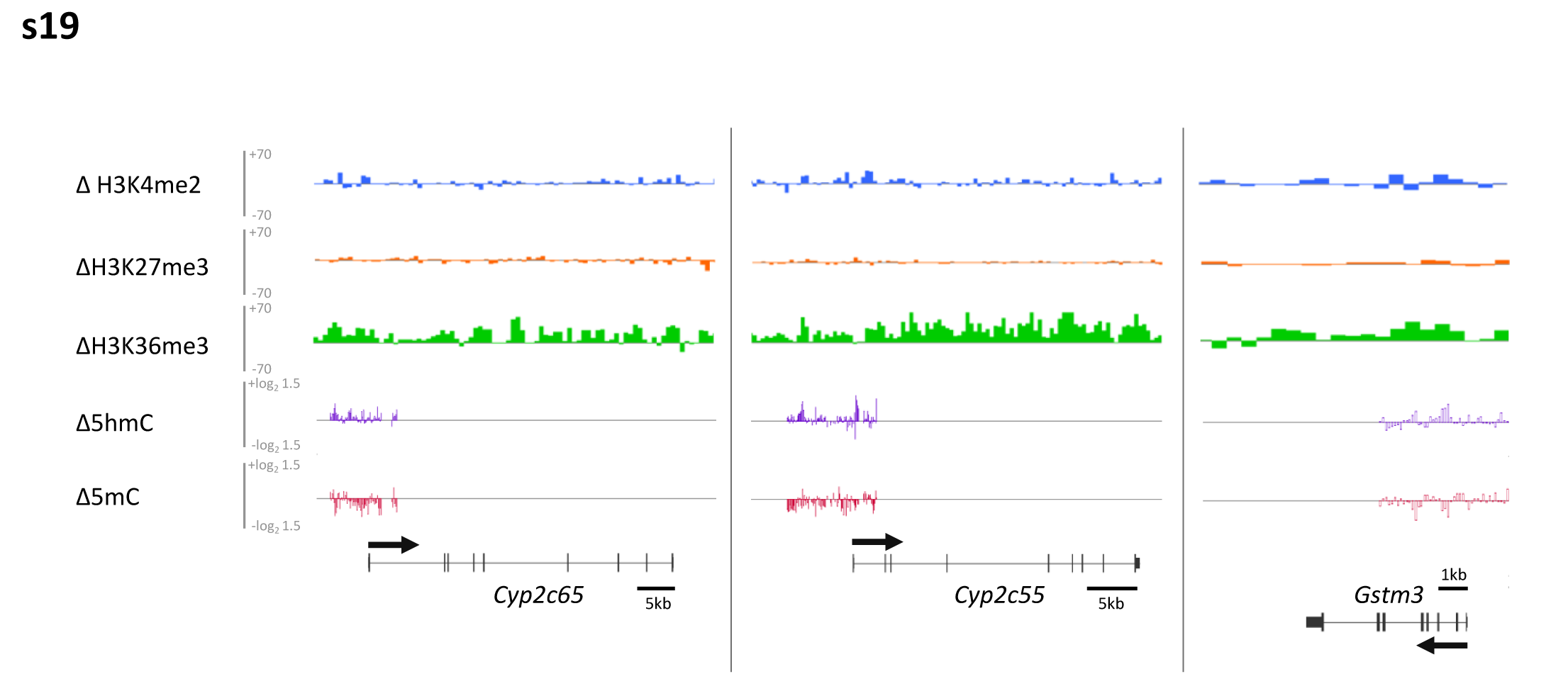

Supplement: Additional file 23 — Figure S19. Examples of changes to the profiles of histone modifications (ChIP-Seq) and DNA modification changes (MeDIP/HmeDIP-chip) across select Cyp2b and Gst genes. Patterns of log2 changes in the signals of 5mC (red), 5hmC (purple), H3K36me3 (green), H3K27me3 (orange) and H3K4me2 (blue) are plotted over these regions. The Y-axis of ChIP-Seq samples were plotted on a scale of +70 to -70+ reads whilst promoter arrays (5hmC and 5mC) plotted from +1.5 log2 to -1.5 log2 values. [file gb-2012-13-10-r93-S23.tiff]

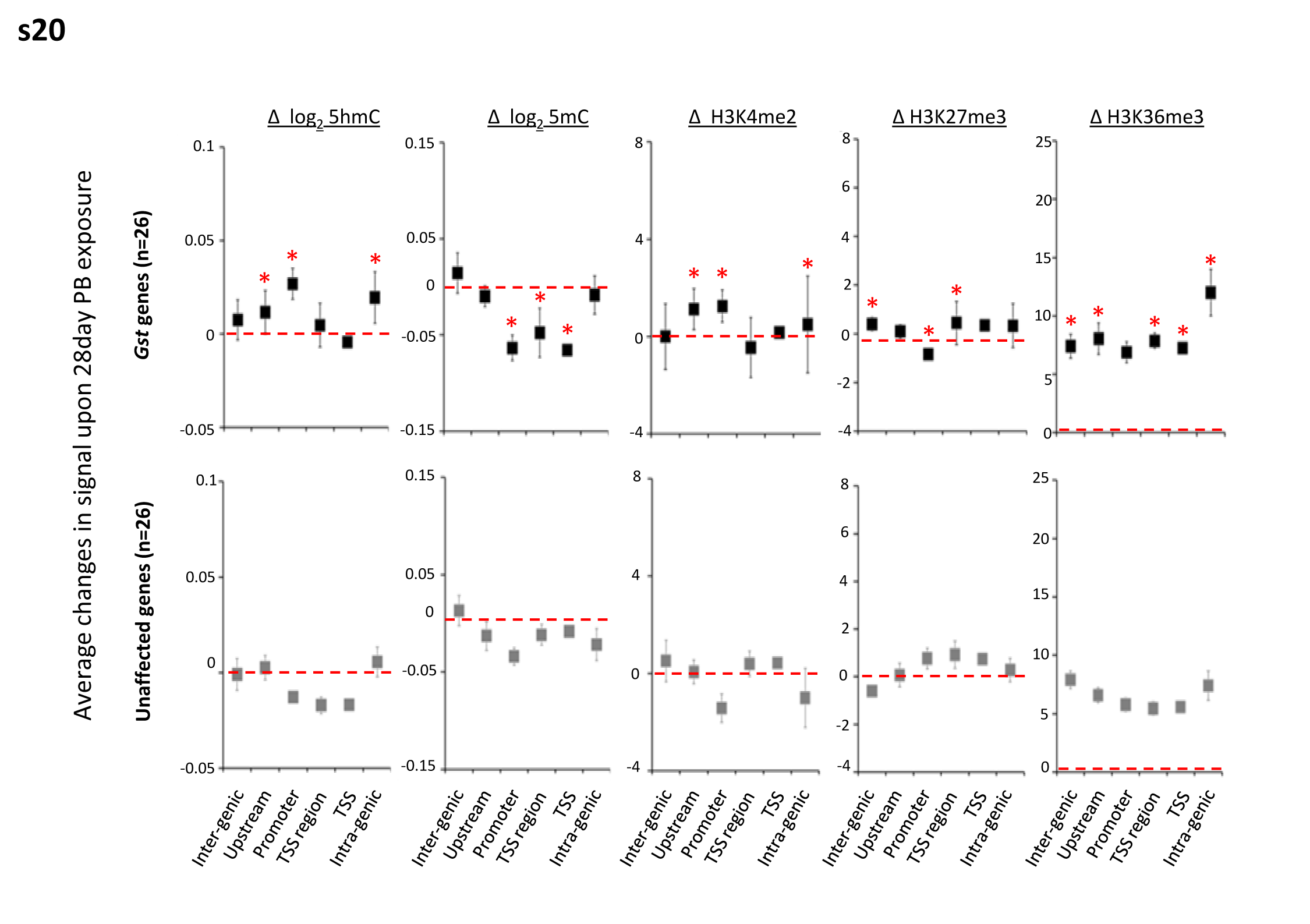

Supplement: Additional file 24 — Figure S20. Plots of average changes in epigenetic marks over Gst gene family in comparison to genes unaffected in their gene expression states following 28-day PB exposure. Average changes in the log2 scores (DNA modifications) or fold change in the number of reads (histone modifications) of the marks are plotted against regions outlined earlier in Figure 1. Error bars are standard error and points showing significant deviation from unaffected genes (Willcox test, P-value < 0.005) are denoted by the red asterisk. Red dotted line represents 0 change in epigenetic mark upon PB exposure. [file gb-2012-13-10-r93-S24.tiff]
