# Supplementary material for: Non-genotoxic carcinogen exposure induces defined changes in the 5-hydroxymethylome
Source: Genome Biol. 2012 Oct 3;13(10):R93. doi: 10.1186/gb-2012-13-10-r93 (PMC3491421; doi:10.1186/gb-2012-13-10-r93)
Supplement: Additional file 10 — Table S1. List of genes with a 5hmC-enriched 'TSS region' (n = 508). [file gb-2012-13-10-r93-S10.doc]

| **List of genes with a 5hmC enriched "TSS region" : n=508** | | | | | | | | | | |
| --- | --- | --- | --- | --- | --- | --- | --- | --- | --- | --- |
| 0610012H03Rik | Alas1 | Cela1 | Ebp | Gp5 | Lcmt1 | Mpdu1 | Plscr2 | Sec16b | Tas1r3 | Zfp52 |
| 1110034G24Rik | Alg1 | Cend1 | Echdc2 | Gpam | Lcp1 | Mpeg1 | Plscr4 | Selk | Tat | Zfp619 |
| 1500011K16Rik | Amn | Cgref1 | Eci1 | Gpd1l | Ldb3 | Mpzl3 | Pnldc1 | Senp7 | Tbc1d10c | Zfp652 |
| 1700001L05Rik | Amot | Chic1 | Eci3 | Gpr110 | Lekr1 | Mrap | Pnma1 | Sepx1 | Tbcd | Zfp709 |
| 1700001P01Rik | Ankrd24 | Clns1a | Efcab4a | Gpr12 | Lingo4 | Mrgpre | Pnp | Serinc4 | Tex19.1 | Zfp819 |
| 1700006E09Rik | Ankrd26 | Cml3 | Ehbp1 | Gpr37l1 | Llph | Mrpl23 | Pnp2 | Serpina12 | Tfpi2 | Zfp874b |
| 1700010I14Rik | Anubl1 | Cno | Eif4e | Gpr84 | Lphn2 | Mrpl24 | Podn | Serpina5 | Thap3 | Znhit2-ps |
| 1700019G17Rik | Apol9b | Cnpy2 | Eif5a2 | Gpx1 | Lrp4 | Mrpl38 | Poglut1 | Serpina9 | Thnsl2 | Zxdc |
| 1700030K09Rik | Arfgef2 | Cntd1 | Elac1 | Gpx7 | Ltc4s | Mrps28 | Pon1 | Serpinb1a | Tie1 |  |
| 1700040L02Rik | Arhgap5 | Cntln | Elane | Grhpr | Mab21l3 | Msh4 | Ppa2 | Sgms2 | Timm13 |  |
| 1700054N08Rik | Arntl2 | Cntnap1 | Eno3 | Grsf1 | Man2a2 | Mstn | Ppp1ca | Sgpp1 | Tmem106a |  |
| 1700074P13Rik | Art3 | Cog1 | Enpp2 | Gsr | Mansc1 | Mtmr1 | Prkar2a | Sirt6 | Tmem136 |  |
| 1700092M07Rik | Asb15 | Col9a2 | Fabp12 | Gstp2 | Map2k1 | Mul1 | Prlr | Slc10a2 | Tmem139 |  |
| 1700102P08Rik | Ascc2 | Coq10a | Fam116b | Gtf2h2 | Map2k4 | Myl7 | Prmt10 | Slc11a1 | Tmem82 |  |
| 1700125D06Rik | Atad5 | Coro6 | Fam123a | Gypc | Mapk15 | Ndst1 | Prok1 | Slc13a2 | Tnfrsf11a |  |
| 2310003F16Rik | Ate1 | Cotl1 | Fam131a | H19 | Mapkap1 | Ndufa5 | Prpf3 | Slc15a5 | Tomm20 |  |
| 2310014L17Rik | Atg4c | Cpa2 | Fam175b | Hdhd2 | Mcm9 | Necap1 | Prrg4 | Slc17a1 | Tomm34 |  |
| 2310022B05Rik | Atoh8 | Crisp2 | Fam187b | Heatr5a | Mdn1 | Neil1 | Prss53 | Slc29a4 | Top2a |  |
| 2410015M20Rik | Atp10d | Crybb3 | Fam26f | Hebp1 | Med21 | Nelf | Psma3 | Slc31a2 | Tpt1p |  |
| 2510003E04Rik | B230120H23Rik | Crygs | Fam82a1 | Hist1h1t | Med28 | Niacr1 | Psmc1 | Slc33a1 | Trak1 |  |
| 2810428I15Rik | B3galt1 | Csrnp1 | Fastkd1 | Hjurp | Mesp2 | Nlrx1 | Ptprcap | Slc35e3 | Trim2 |  |
| 3110003A17Rik | B3galt4 | Cst3 | Fat1 | Hlcs | Mettl10 | Nmi | Ptprf | Slc37a1 | Trmt11 |  |
| 4632415K11Rik | B3gnt9-ps | Ctcfl | Fbp2 | Iah1 | Mfap1b | Nnmt | Pycr2 | Slc39a14 | Trnt1 |  |
| 4833422F24Rik | Baalc | Ctif | Fbxl22 | Ifi27l1 | Mff | Nol11 | R3hdml | Slc3a1 | Tspan10 |  |
| 4833442J19Rik | Bbs2 | Ctsk | Fgfr2 | Igfbp6 | Mfsd1 | Noxa1 | Rab34 | Slc45a2 | Tspyl5 |  |
| 4921513D23Rik | Bcl7b | Cyp17a1 | Fitm1 | Il10rb | Mfsd2a | Npc1l1 | Rab9 | Slc7a9 | Tssk3 |  |
| 5430437P03Rik | Birc3 | Cyp2b10 | Fkbpl | Il1rn | Mga | Nsd1 | Rad18 | Smc1b | Tti1 |  |
| 5830433M19Rik | Brd1 | Cyp2c44 | Fnta | Inpp5f | Mgll | Nsmce1 | Rassf8 | Smpdl3a | Ttll4 |  |
| 6330416G13Rik | Btbd19 | Cyp2j9 | Foxn3 | Iqce | Mia1 | Numb | Rcbtb2 | Smyd1 | Ttpa |  |
| A1bg | Butr1 | Cyp7b1 | Fpgs | Isoc2b | Mios | Nupr1 | Rdh1 | Snhg3 | Twistnb |  |
| A230065H16Rik | C1qtnf9 | Cys1 | Frrs1 | Ispd | Mir141 | Ociad1 | Rhbdd1 | Snora31 | Uhmk1 |  |
| A730008H23Rik | C2cd4d | D9Ertd402e | Fscn3 | Itgb1bp2 | Mir1934 | Oit3 | Ribc2 | Snx27 | Ushbp1 |  |
| Aars | C4a | Dap | Furin | Itgb2 | Mir1956 | Olr1 | Rilpl1 | Sox15 | Usp16 |  |
| Abca9 | Calr3 | Dbn1 | Fut11 | Josd2 | Mir200c | Otub1 | Rnase10 | Sox30 | Usp19 |  |
| Abcc3 | Capn3 | Dbr1 | Gabbr1 | Jund | Mir23b | Oxsr1 | Rnase13 | Spint2 | Usp49 |  |
| Acaa1b | Car15 | Dcaf4 | Gatad2b | Kbtbd13 | Mir24-1 | Pank3 | Rpl21 | Stambpl1 | Utrn |  |
| Acacb | Card6 | Dcxr | Gigyf1 | Kcnk5 | Mir27b | Paox | Rpl8 | Stat1 | Vcpip1 |  |
| Accn3 | Casp7 | Ddx17 | Gimap4 | Kidins220 | Mir29a | Park7 | Rpp21 | Stk38l | Vps13d |  |
| Acot11 | Cbx1 | Decr1 | Gkn3 | Klf12 | Mir29b-1 | Parp11 | Rps24 | Ston1 | Wdr60 |  |
| Acot4 | Ccdc11 | Defb36 | Gm1006 | Klhdc7b | Mir3074-1 | Parp16 | Rps6kl1 | Stx5a | Wdsub1 |  |
| Acrbp | Ccdc150 | Dhrs9 | Gm11744 | Klhdc9 | Mir30a | Pde6c | Rras2 | Sucla2 | Zbtb4 |  |
| Actr8 | Ccdc56 | Dhx30 | Gm13939 | Klhl5 | Mir30c-2 | Pef1 | Rtdr1 | Sult5a1 | Zbtb40 |  |
| Adam2 | Ccdc79 | Dkk4 | Gm15348 | Klk15 | Mir31 | Pgam5 | Rtp4 | Sumf2 | Zdhhc23 |  |
| Adcy6 | Ccdc91 | Dkkl1 | Gm52 | Kpna2 | Mir484 | Pgbd1 | S100a16 | Sumo2 | Zfand2a |  |
| Adh6a | Ccr10 | Dnajc10 | Gm711 | L3mbtl2 | Mir802 | Pgm2 | Sag | Surf4 | Zfp202 |  |
| Adh7 | Cd160 | Dnajc27 | Gm732 | Lace1 | Mir804 | Pik3ca | Scarb1 | Sycp3 | Zfp275 |  |
| AI317395 | Cd1d1 | Dnm3 | Gm839 | Lactb | Mirlet7b | Pion | Scgb1a1 | Synpo | Zfp295 |  |
| AI428936 | Cd276 | Dpep2 | Gm889 | Lactb2 | Mirlet7c-2 | Pir | Scp2 | Taar1 | Zfp35 |  |
| Ak1 | Cd68 | Dpp4 | Gne | Lag3 | Mknk2 | Pld6 | Sdr42e1 | Tagln | Zfp422 |  |
| Ak4 | Cd9 | Dsn1 | Gng11 | Lamp1 | Morc3 | Plscr1 | Sebox | Tap2 | Zfp518a |  |

**Supplementary Table 1.**

List of genes identified as containing an enrichment of 5hmC (region is associated with at least one peak of 5hmC - see materials and methods)over the TSS region (TSS+/-250bp) in normal mouse livers.
