# Supplementary material for: Non-genotoxic carcinogen exposure induces defined changes in the 5-hydroxymethylome
Source: Genome Biol. 2012 Oct 3;13(10):R93. doi: 10.1186/gb-2012-13-10-r93 (PMC3491421; doi:10.1186/gb-2012-13-10-r93)
Supplement: Additional file 11 — Table S2. Average promoter 5hmC and 5mC log2 scores across all the genes on the array. [file gb-2012-13-10-r93-S11.doc]

|  |  | **Average Promoter 5hmC signal (log2)** | **Average Promoter 5mC signal (log2)** | **Average Relative Expression score (log2)** |
| --- | --- | --- | --- | --- |
| **All genes** |  | 0.028 | -0.099 | 6.410 |
| **5hmC enriched TSS genes** |  | 0.650 * | 0.020 * | 4.264 * |

**Supplementary Table 2.** Average promoter 5hmC and 5mC log_2_ scores across all the genes on the array compared to genes with 5hmC enriched TSS regions (n=508). Average log2 expression scores for control liver are also shown. Significant P-values (Willcox test <0.001) are denoted by an asterisk (*). Genes with 5hmC enriched TSS regions contain far greater levels of both 5hmC and 5mC over their promoters and are also significantly reduced in their relative expression scores (log2 Affymetrix normalised RMA expression score, see Materials and methods).
