# Supplementary material for: Non-genotoxic carcinogen exposure induces defined changes in the 5-hydroxymethylome
Source: Genome Biol. 2012 Oct 3;13(10):R93. doi: 10.1186/gb-2012-13-10-r93 (PMC3491421; doi:10.1186/gb-2012-13-10-r93)
Supplement: Additional file 21 — Table S3. Top 25 genes induced upon 28-day PB exposure. [file gb-2012-13-10-r93-S21.doc]

| **Top 25 genes induced upon 28day PB exposure** | | | | | | |
| --- | --- | --- | --- | --- | --- | --- |
| **gene name** | **Mean Log2**  **PPR TSS Δ 5hmC** | **Mean Log2**  **PPR Δ 5mC** | **Mean Log2**  **PPR**  **Δ H3K4me2** | **Mean Log2**  **gene body**  **Δ H3K27me3** | **Mean Log2**  **gene body**  **Δ H3K36me3** | **Δ log2 fold change expression** |
| Cyp2b10* | 0.04 | -0.56 | 1.81 | -2.32 | 1.64 | 7.32 |
| Cyp2c65* | 0.07 | -0.17 | 0.29 | -0.56 | 0.80 | 6.86 |
| Cyp2c55* | 0.09 | -0.12 | 0.49 | -0.36 | 1.52 | 4.15 |
| Cyp2b9* | 0.05 | -0.13 | 1.93 | -0.05 | 0.81 | 4.14 |
| Gstm3* | 0.10 | 0.01 | 0.40 | -0.17 | 1.43 | 3.37 |
| Pnliprp1 | 0.01 | -0.07 | 0.22 | 0.02 | 0.78 | 3.26 |
| Akr1b7 | 0.04 | 0.01 | 0.26 | -0.13 | 0.85 | 3.23 |
| Cyp26a1* | -0.03 | 0.00 | -0.13 | 0.20 | 0.83 | 2.89 |
| 1700067K01Rik | 0.07 | 0.04 | -0.71 | 0.46 | 0.51 | 2.83 |
| Gstt3* | 0.08 | 0.00 | 1.05 | -0.50 | 0.91 | 2.71 |
| Orm3 | 0.02 | -0.03 | 2.10 | 0.26 | 0.75 | 2.42 |
| Orm2 | -0.03 | 0.10 | 0.53 | 0.15 | 0.89 | 2.41 |
| Lect1 | 0.03 | 0.04 | 0.87 | -0.29 | 0.90 | 2.24 |
| Alas1 | 0.06 | -0.07 | 0.09 | 0.99 | 1.46 | 2.24 |
| Hspa1a | 0.08 | 0.02 | 0.40 | -1.07 | 0.78 | 2.23 |
| Meig1 | 0.05 | -0.12 | -0.64 | -0.32 | 0.32 | 2.16 |
| Abcc4 | 0.02 | 0.01 | 0.05 | -0.17 | 0.82 | 2.15 |
| Wisp1 | 0.01 | 0.15 | -0.68 | -0.23 | 0.53 | 2.13 |
| Cyp2b13* | 0.11 | -0.14 | 0.29 | -0.02 | 1.35 | 1.94 |
| Tsku | -0.01 | -0.11 | 0.74 | -0.05 | 1.30 | 1.92 |
| Kcnk1 | 0.01 | 0.00 | -0.83 | 0.05 | 0.62 | 1.88 |
| Cyp2c37* | 0.02 | -0.24 | 0.51 | -1.20 | 1.21 | 1.85 |
| Fam171a1 | 0.03 | 0.03 | 0.15 | -0.08 | 0.68 | 1.82 |
| Por | -0.01 | 0.00 | 0.51 | -0.04 | 1.26 | 1.78 |
| Krt4 | 0.06 | 0.11 | 0.17 | -0.03 | 1.27 | 1.77 |
| **AVE Induced** | **0.039** | **-0.05** | **0.395** | **-0.218** | **0.969** | **2.87** |
| **AVE genome** | **0.003** | **-0.015** | **-0.321** | **0.123** | **0.767** | **-0.004** |
| **P value** | **3.21E-03** | **1.39E-03** | **1.60E-04** | **4.48E-04** | **5.08E-04** | **1.21E-05** |

**Supplemental Table 3**

Both DNA and histone modifications are altered in the 25 most induced genes displaying following 28day PB exposure. Alongside the log_2_ fold changes in gene expression levels, the average log2 changes in promoter proximal regions ("PPRs": TSS+/-1kb) 5hmC,5mc and H3K4me2 levels and average changes in gene body H3K27me3 and H3K36me3 levels are shown. These marks differ significantly when compared to the average levels of change of these marks throughout the genome.
