# Supplementary material for: Non-genotoxic carcinogen exposure induces defined changes in the 5-hydroxymethylome
Source: Genome Biol. 2012 Oct 3;13(10):R93. doi: 10.1186/gb-2012-13-10-r93 (PMC3491421; doi:10.1186/gb-2012-13-10-r93)
Supplement: Additional file 22 — Table S4. Gene Ontology terms for genes induced following PB exposure. [file gb-2012-13-10-r93-S22.doc]

| GO analysis: genes Induced >1.5 fold | Number of genes | **p-value** | **FDR** |
| --- | --- | --- | --- |
| oxidative reduction | 9 | 2.52E-06 | 1.40E-05 |
| fatty acid metabolic process | 4 | 1.12E-01 | 5.60E+01 |
| fatty acid biosynthetic process | 3 | 5.70E-02 | 5.60E+01 |
| Response to drug | 3 | 3.76E-02 | 6.10E+01 |
|  |  |  |  |
|  |  |  |  |
|  |  |  |  |

**Supplemental Table4**

Go terms for genes >log_2_ 1.5 fold induced following PB exposure. Upregulated genes appear to be those associated with oxidative reduction (including the *Cyp* genes) as well as response to drugs. FDR = False discovery rate. P-value was generated using DAVID bioinformatics resources (http://david.abcc.ncifcrf.gov/) based on a modified Fisher Exact calculation.
