# Supplementary material for: Non-genotoxic carcinogen exposure induces defined changes in the 5-hydroxymethylome
Source: Genome Biol. 2012 Oct 3;13(10):R93. doi: 10.1186/gb-2012-13-10-r93 (PMC3491421; doi:10.1186/gb-2012-13-10-r93)
Supplement: Additional file 25 — Table S5. List of DNA primers used in this study. [file gb-2012-13-10-r93-S25.doc]

| **qPCR** | forward | Reverse | chr | start | stop | midpoint |
| --- | --- | --- | --- | --- | --- | --- |
| Tex19.1 promoter | GGGAGATATGTAAATGAGCTGG | CATCCTTACCTCCCTGACTGAG | chr11 | 121007395 | 121007545 | 121007470 |
| actb promoter | CGAGCACTTAAGTGGATGA | GCTTTCCGGCTATTGCTAG | chr5 | 143668797 | 143668940 | 143668869 |
| Gapdh promoter | CCACTCCCCTTCCCAGTTTC | CCTATAAATACGGACTGCAGC | chr6 | 125115507 | 125115654 | 125115581 |
| Cyp2b10 upstream | TGGTTGATTCTCCCACCTTC | ATGTCCTCTGGTGAGCATCC | chr7 | 26674441 | 26674558 | 26674500 |
| Cyp2b10 promoter 5' | GGAACCTCTTGCAGATGGAC | AGGGGGAAACCCACACTAAG | chr7 | 26682825 | 26682913 | 26682869 |
| Cyp2b10 promoter 3' | TCCTTCCACGGTGTAGGTTC | CCACACAACTCCAACGTAGC | chr7 | 26682955 | 26683071 | 26683013 |
| H19 promoter | GCCAAGAGAGAAGAAGGAGA | GAATGTTGAAGGACTGAGGG | chr7 | 149763644 | 149763930 | 149763787 |
| Cyp2b10_Intron1_3’ | TCCTTCCACGGTGTAGGTTC | CCACACAACTCCAACGTAGC | chr7 | 26683050 | 26683069 | 26683060 |
| H19 ICR | GCATCTGAACGCCCCAATTA | GCATGGTCCTCAAATTCTGCA | chr7 | 149767303 | 149767431 | 149767367 |
| Gnas ICR | CAACGAGCCTGCCCAGGGAGAC | CTGTAGTCTTCTCGGAAGCCAG | chr2 | 174,123,869 | 174,124,018 | 174123944 |
| Csa promoter | TGGTTGGCATTTTATCCCTAGAAC | GCAACATGGCAACTGGAAACA | chr11 | 95,277,372 | 95,277,505 | 95277439 |
| Ccdc34 promoter | CAGCAGTGTCTCTCTCCTTCC | CAATGGCCTCATCAACTGTG | chr2 | 109,860,823 | 109,860,951 | 109860887 |
| Tyms prmoter | TGCAAGGGGAAAAGGCTAAG | GGTGATGGAAGGTTTTGCTG | chr5 | 30,408,108 | 30,408,254 | 30408181 |
| Cbx2 promoter | TTTACAAGCCCTGCAGATCC | GAGGACCCAAGCCTAAAACC | chr11 | 118,901,185 | 118,901,277 | 118901231 |
| Pou3f4 promoter | ATGGCGAAAGATCCTCTCTG | AGGGGAGAAAACCTTGCATC | chrX | 108,007,867 | 108,007,957 | 108007912 |
| Tacr3 promoter | AGCACAGTTGGAGAGACTGG | GAGGCAGGTGTTGAAAGGAG | chr3 | 134,487,028 | 134,487,150 | 134487089 |
|  |  |  |  |  |  |  |
|  |  |  |  |  |  |  |
|  |  |  |  |  |  |  |
|  |  |  |  |  |  |  |
| **EpiMark qPCR** | forward | Reverse | chr | start | stop | midpoint |
| non genic: hmc-ve (chr7:149709621) | CCTATCTTTGTCTGTCCAGC | CTCCCTGAACCTCTCATTCATC | chr7 | 149709621 | 149709863 | 149709742 |
| upstream region hmc+ve (Cyp2b10) | TTTTGAGATGGGGTCTGTCC | GGATGTTGGCTTGACCTTTC | chr7 | 26683571 | 26683909 | 26683740 |
| TSS: hmc +ve (Tspan10) | GGCTAGAAAGACCAGATGTG | GAGCTAGGCCATTAAGTGTC | chr11 | 120303858 | 120304158 | 120304008 |
| TSS: hmc +ve (H19) | GCCAAGAGAGAAGAAGGAGA | GAATGTTGAAGGACTGAGGG | chr7 | 149763644 | 149763930 | 149763787 |
| TSS: hmc -ve (Gapdh) | CCACTCCCCTTCCCAGTTTC | CCTATAAATACGGACTGCAGC | chr6 | 125115507 | 125115654 | 125115581 |
| Genic: hmc+ve (Gstm3 exonic) | AGAGGGGTGATAAAGAAGGG | AAAGTCAGAGAGACAGGTGG | chr3 | 107771265 | 107771474 | 107771370 |
| Genic: hmc+ve (Gstt3 exonic) | CCCACTCTTTTCCATCCATC | CTCAACAATTCCTTCCACGG | Chr10 | 75238846 | 75239093 | 75238970 |

**Supplemental Table5**

List of primers used in this study.Sequences and coordinates for primers used in standard qPCR and Epimark specific qPCR (amplicons contain a single *MspI* cut site) are shown.
